# RGB Thermally Activated Delayed Fluorescence Emitters for Organic Light‐Emitting Diodes toward Realizing the BT.2020 Standard

**DOI:** 10.1002/advs.202303504

**Published:** 2023-08-16

**Authors:** Xiaochun Fan, Xiaoyao Hao, Feng Huang, Jia Yu, Kai Wang, Xiaohong Zhang

**Affiliations:** ^1^ Institute of Functional Nano & Soft Materials (FUNSOM) Soochow University Suzhou Jiangsu 215123 P. R. China; ^2^ Jiangsu Key Laboratory of Advanced Negative Carbon Technologies Soochow University Suzhou Jiangsu 215123 P. R. China; ^3^ Jiangsu Key Laboratory of Carbon‐Based Functional Materials and Devices Soochow University Suzhou Jiangsu 215123 P. R. China

**Keywords:** BT. 2020 standard, donor‐acceptor‐type, multiple resonance, organic light‐emitting diodes, spectral narrowing, spectral shifting, thermally activated delayed fluorescence

## Abstract

With the surging demand for ultra‐high‐resolution displays, the International Telecommunication Union (ITU) announce the next‐generation color gamut standard, named ITU‐R Recommendation BT.2020, which not only sets a seductive but challenging milestone for display technologies but also urges researchers to recognize the importance of color coordinates. Organic light‐emitting diodes (OLEDs) are an important display technology in current daily life, but they face challenges in approaching the BT.2020 standard. Thermally activated delayed fluorescence (TADF) emitters have bright prospects in OLEDs because they possess 100% theoretical exciton utilization. Thus, the development of TADF emitters emitting primary red (R), green (R), and blue (B) emission is of great significance. Here, a comprehensive overview of the latest advancements in TADF emitters that exhibit Commission Internationale de l'Éclairage (CIE) coordinates surpassing the National Television System Committee (NTSC) and approaching BT.2020 standards is presented. Rational strategies for molecular designs, as well as the resulting photophysical properties and OLED performances, are discussed to elucidate the underlying mechanisms for shifting the CIE coordinates of both donor‐acceptor and multiple resonance (MR) typed TADF emitters toward the BT.2020 standard. Finally, the challenges in realization of the wide‐color‐gamut BT.2020 standard and the prospects for this research area are provided.

## Graphical abstract

Ultra‐high‐resolution is an inevitable trend for future high‐end displays based on organic light‐emitting diode (OLED) technology. Here, recent advancements in red/green/blue (RGB) OLEDs using thermally activated delayed fluorescence (TADF) emitters aimed at the BT.2020 standard are summarized. Based on elucidating the underlying mechanisms for shifting the Commission Internationale de l'Éclairage (CIE) coordinates, the challenges and prospects are further discussed.

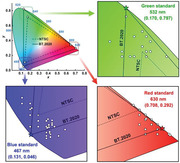

## 1 Introduction

Organic light‐emitting diode (OLED) display technology has become an integral part of our daily life, and its widespread applications encompass smartphones, high‐end televisions, and augmented/virtual reality (AR/VR) devices, as it offers several attractive features such as a high contrast ratio, wide viewing angle, fast refresh rate, brilliant color rendition, and design flexibility.^[^ ^1^, ^2^, ^3^ ^]^

When technically evaluating the performance of a display item, a wide color gamut is invariably one of the most significant characteristics that needs to be taken into consideration.^[^ ^4^, ^5^ ^]^ As an electrically‐powered, self‐illuminating technology, OLEDs generally utilize primary RGB (i.e., red (R), green (G), and blue (B)) subpixel devices to reproduce the brightness and color of each pixel. The color gamut of OLED displays is typically an enclosed area determined by the Commission Internationale de l'Éclairage (CIE) coordinates of the primary RGB subpixel devices in the CIE coordinate system.^[^ ^6^, ^7^ ^]^ In the widely accepted National Television System Committee (NTSC) standard, the CIE coordinates of the primary RGB should be located at (0.67, 0.33), (0.21, 0.71), and (0.14, 0.08), respectively.^[^ ^8^ ^]^ After continuous innovative development in recent decades, commercial OLED displays can already achieve sufficient RGB color purities that meet the NTSC standard. However, people's pursuit of higher quality displays is endless. In 2012, the International Telecommunication Union (ITU) announced the next‐generation color gamut standard—ITU‐R Recommendation BT.2020 (i.e., Rec.2020)—to keep up with the increasing demand for ultra‐high‐resolution displays.^[^ ^9^ ^]^ Compared to the NTSC standard, the BT.2020 standard allows for a 1.5‐fold improvement in color gamut, which covers ≈75.8% of the entire CIE 1931 color space chromaticity diagram. Such a large expansion of the color gamut derives from the redefinition of the CIE coordinates of primary RGB colors, i.e., (0.708, 0.292) for red, (0.170, 0.797) for green, and (0.131, 0.046) for blue. To approach this leading‐edge standard, developing high color purity OLED emitters is a critical issue.

Among the current OLED emitters, thermally activated delayed fluorescence (TADF) emitters are promising alternative candidates for commercialized noble‐metal‐containing phosphors, since they theoretically possess 100% internal quantum efficiency (IQE) with cheaper pure organic chemical structures.^[^ ^10^ ^]^ A key dynamic process of the TADF mechanism, that is, triplet‐to‐singlet reverse intersystem crossing (RISC), requires a tiny singlet‐triplet splitting energy (ΔE_ST_). It can be realized via the spatial separation of the frontier molecular orbital (FMO) distribution. Thus, twisted or space‐confined donor‐acceptor (D‐A) molecular design was proposed early and has been repeatedly proven to be a facile strategy for enabling TADF.^[^ ^11^, ^12^, ^13^ ^]^ In addition, to date, by judiciously modulating the push‐pull capacity, linking position, number of diverse π‐conjugated donors and acceptors, etc., full‐color TADF emitters have been explored, and some of them have already achieved external quantum efficiencies (EQEs) of over 30% in OLEDs.^[^ ^14^, ^15^, ^16^, ^17^, ^18^ ^]^ However, because of the long‐range charge transfer (CT) nature, loose molecular structures, and strong vibronic coupling between the ground state (S_0_) and the first singlet excited state (S_1_), D‐A‐type TADF emitters often induce broad electroluminescence (EL) spectra with full widths at half maxima (FWHM) of over 70 nm.^[^ ^19^, ^20^ ^]^ The corresponding low color purity is apparently a terrible weakness of D‐A‐type TADF emitters, strongly hindering them from approaching the BT.2020 standard. Despite this weakness, it is worth noting that some D‐A‐type emitters with peaks in the deep‐blue and deep‐red regions have been reported to approach the blue and red standards of BT.2020. This is due to the special edge‐position of blue and red colors in the visible spectrum.

To overcome the aforementioned drawbacks of conventional D‐A‐type TADF emitters, Hatakeyama et al. pioneered a TADF molecular design concept based on the multiple resonance (MR) effect.^[^ ^21^ ^]^ Specifically, the MR effect is induced by the opposite resonance features of electron‐deficient and electron‐rich atom or group pairs (e.g., boron/nitrogen (B/N) and N/carbonyl), which results in alternating localization of the highest occupied molecular orbital (HOMO) and the lowest unoccupied molecular orbital (LUMO) in rigid polycyclic frameworks. Such a finely arranged FMO distribution successfully endows MR emitters with small ΔE_ST_ values (generally below 0.2 eV), thus leading to obvious TADF characteristics. More importantly, benefiting from the dynamic structural rigidity change, structural relaxations, vibronic couplings between the S_1_‐S_0_ transitions, and stretching/scissoring vibrations in MR emitters are all well suppressed, leading to a narrowband emission with a small FWHM.^[^ ^22^ ^]^ The above two features not only ensure high performance in emission efficiency but also break through the color purity limitation in conventional D‐A‐type TADF emitters. Since the first report on an MR‐TADF emitter (designated **DABNA‐1**) in 2016, significant efforts have been devoted to this new paradigm, which greatly accelerates the step of RGB TADF OLEDs to meet the BT.2020 standard.^[^ ^23^, ^24^, ^25^ ^]^

In this review, we summarize the recent advances in RGB TADF emitters that exhibit electroluminescent colors exceeding the NTSC and close to the BT.2020 standards in CIE coordinates. Under the general heading of each primary RGB color, we further categorize these emitters and the corresponding OLEDs based on their original molecular design concepts by grouping them into D‐A‐type and MR‐type TADF emitters. Rational molecular design strategies, as well as the resulting photophysical properties and OLED performance, are discussed to understand the underlying mechanisms for shifting the CIE coordinates of both D‐A‐type and MR TADF emitters to the BT.2020 standard. Finally, we provide our perspective on the remaining challenges in this research area that need be overcome to develop the next generation of wide‐color gamut OLED displays.

## 2 Basic Principle for Shifting CIE Coordinates to the BT.2020 Standard

**Figure 1:**
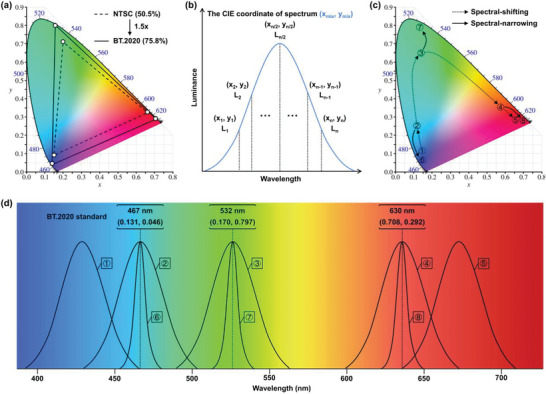
a) Comparison of NTSC and BT.2020 in the CIE 1931 color space chromaticity diagram; b) schematic diagram of combined CIE coordinates; c) shift of CIE coordinates corresponding to d) spectral shifting/narrowing in the whole visible spectrum.

The CIE 1931 color space is the first defined quantitative relationship between the distribution of emission wavelengths in the visible spectrum and physiologically perceived colors in human color vision.^[^ ^26^ ^]^ To be precise, a full plot of all visible colors should be a 3D space diagram (i.e., CIE XYZ color cube) since the human eye has three types of color sensors that respond to different ranges of emission wavelengths.^[^ ^27^, ^28^ ^]^ In this case, a monochromatic color needs three parameters to define, where X/Z are responsible for chromaticity, and Y is the luminance. To allow the convenience of the CIE concept in scientific work, the researchers further devised a luminance‐chromaticity color space based on the CIE XYZ color cube, called the CIE 1931 color space chromaticity diagram. The derived color space is specified by coordinates *x* and *y*, which can be described using the following expression:^[^ ^29^ ^]^

(1) $$x\;=\frac{X}{X+Y+Z}\;$$

(2) $$y\;=\frac{Y}{X+Y+Z}\;$$

where *x* and *y* simply represent the red and green components, respectively. With this ingenious conversion, a monochromatic color can be simplified by using two parameters for definition. Note that these two parameters carry the same information as CIE XYZ, indicating the feasibility of defining all chromaticities visible to human eyes.^[^ ^30^, ^31^ ^]^ As depicted in **Figure** **1**, the outer curved boundary of the CIE 1931 color space chromaticity diagram is the spectral locus with emission wavelengths shown in nanometers, and the point located in this spectral locus is the standard CIE coordinate at the corresponding wavelength. For instance, the CIE coordinates of the primary RGB colors of the BT.2020 standard originate from wavelengths at 630, 532, and 467 nm, respectively.

However, due to the inevitable structural vibrations, an actual emissive spectrum always covers an integrated region rather than a sharp line in a specific wavelength in the visible spectrum.^[^ ^32^ ^]^ Therefore, the CIE coordinates of an actual emissive spectrum should be evaluated by the following mixed‐model formulas:^[^ ^29^, ^33^ ^]^

(3) $$x_{mix}=\frac{\frac{x_{1}}{y_{1}}L_{1}+\frac{x_{2}}{y_{2}}L_{2}+\cdots +\frac{x_{n}}{y_{n}}L_{n}}{\frac{L_{1}}{y_{1}}+\frac{L_{2}}{y_{2}}+\cdots +\frac{L_{n}}{y_{n}}}\;$$

(4) $$y_{mix}=\frac{L_{1}+L_{2}+\cdots +L_{n}}{\frac{L_{1}}{y_{1}}+\frac{L_{2}}{y_{2}}+\cdots +\frac{L_{n}}{y_{n}}}\;$$

where (*x*_mix_, *y*_mix_) are the CIE coordinates of the resulting color; (*x*_1_, *y*_1_), (*x*_2_, *y*_2_), …, (*x*_n_, *y*_n_) are the CIE coordinates of the mixture components; and L_1_, L_2_, …, L_n_ are their corresponding luminance (Figure 1b). According to the above formulas, the shift trajectory of the CIE coordinates corresponding to the gradual translation of an amorphous spectrum from the blue to the red region can be roughly simulated (Figure 1c). Hence, while the CIE coordinates of an amorphous spectrum peaking at 467 nm are far from the BT.2020 blue standard, there is a feasible means to approach the BT.2020 blue standard, i.e., a wide range of spectral blueshifts (e.g., from ① to ② in Figure 1c,d). Likewise, a wide range of spectral redshifts can also be utilized for an amorphous spectrum peaked at 630 nm to approach the BT.2020 red standard (e.g., from ④ to ⑤ in Figure 1c & d). In sharp contrast, shifting a green amorphous spectrum only plays a limited role in adjusting the corresponding CIE coordinates to approach the BT.2020 green standard, because the green band is between the blue and red bands in the whole visible region. Fortunately, the mixed‐model formulas also give another more efficient way to shift the CIE coordinates, i.e., narrow spectral bandwidth. As depicted in Figure 1d, since the contribution of a peak wavelength gradually increases when the spectrum becomes tighter, a narrower amorphous spectrum invariably shows higher quality CIE coordinates than wider ones peaked at the same wavelength.^[^ ^32^ ^]^ More importantly, this method is universally available throughout the entire visible range, thus bringing hope for RGB OLEDs to realize the BT.2020 standard.

Depending on the molecular design concept, current TADF emitters can be mainly classified into two material types: conventional D‐A‐type and MR‐type TADF emitters. These two types of materials may demonstrate similar effectiveness in term of their TADF properties, which allows them to achieve ≈100% exciton utilization in OLED applications. However, their emissive spectra are quite dissimilar due to their obvious differences in terms of molecular structural rigidities and FMO distributions. Conventional D‐A‐type TADF emitters with long‐range CT features always show amorphous broad emissions, whereas MR‐type TADF emitters generally exhibit narrowband emissions. Therefore, for developing conventional D‐A‐type TADF emitters that approach the BT.2020 standard, a dominant method is spectral shifting, which can be realized by modulation of the optical bandgaps (e.g., combination of donors and acceptors with different electron‐donating and electron‐accepting abilities). For MR‐type TADF emitters, except for spectral shifting, narrowing emission spectra is also a feasible pathway (usually both are done at the same time), where commonly applied molecular design strategies include peripheral/core modification, π‐conjugate extension, and atomic distributed adjustment. To date, the currently developed TADF emitters with CIE coordinates exceeding the NTSC and close to the BT.2020 standard are mainly concentrated in the red/blue region, while in the green region, only a few MR‐type TADF emitters are adequate. The following sections will provide an overview of these RGB OLED emitters.

## 3 TADF Emitters toward the BT.2020 Red Standard

**Figure 2:**
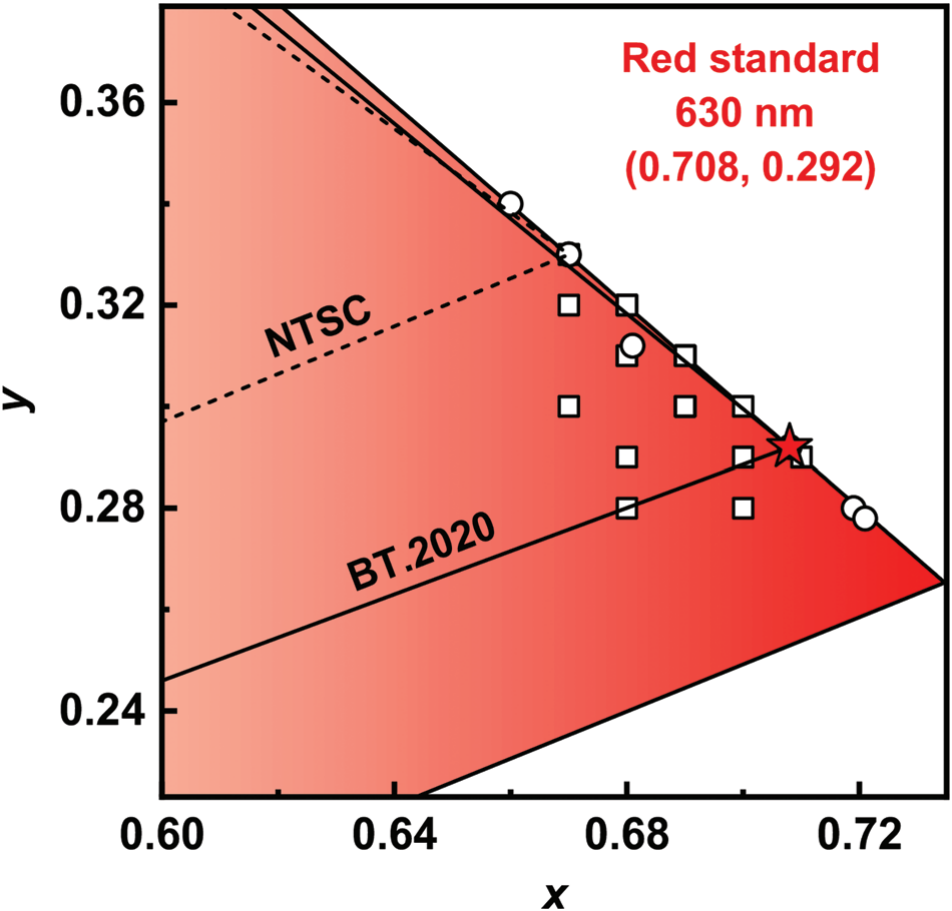
The CIE coordinates of all the summarized D‐A‐type (square) and MR‐type (circle) TADF emitters in the red region.

According to the mixed‐model formulas of CIE coordinates, the BT.2020 red standard can theoretically be realized by sufficiently redshifting the spectra because the red region is located at the far‐right end of the whole visible spectrum. This means that D‐A‐type TADF emitters with broad emission characteristics may tune their corresponding CIE coordinates close to the BT.2020 red standard, but only with extremely deep‐red emissions. On the other hand, for MR‐type TADF emitters with excellent narrowband emission features, only a small redshift is required to shift their CIE coordinates close to the BT.2020 red standard. In this section, two types of red TADF emitters are both summarized, and strategies to shift the CIE coordinates close to the BT.2020 red standard are discussed (**Figure** **2**, **Table** **1** **and** **2**).

**Table 1:** PL and EL data of D‐A‐type red TADF emitters discussed in Section 3.1

Emitter	τ_D_ ^a)^ [µs]	λ_EL_ ^b)^ [nm]	EQE_max/100/1000_ ^c)^ [%]	CE_max_ ^d)^ [cd A^−1^]	PE_max_ ^e)^ [lm W^−1^]	Lifetime ^[h]^	CIE (x, y)^f)^	Reference
TPA‐QCN	201	700	9.4/0.9/0.6	1.6	1.6	‐	(0.68, 0.31)	[36]
	0.80	728 ^g)^	3.9/0.09/‐^g)^	0.3 ^g)^	0.3 ^g)^	‐	(0.69, 0.31)^g)^	[36]
*p*CNQ‐TPA	14.0	660	30.3/8.5/4.9	13.4	13.8	‐	(0.69, 0.31)	[37]
TPA‐Ph‐DBPzDCN	297	708	5.5/1.9/‐	0.61	‐	‐	(0.69, 0.30)	[38]
TPA‐PPDCN	2.0	692	16.4/4.3/1.1	3.1	2.7	‐	(0.70, 0.30)	[39]
TPA‐DCCP	86.2	668	9.8/3.1/1.2	4.0	4.0	‐	(0.68, 0.32)	[40]
	0.76	708 ^g)^	2.1/0.9/‐^g)^	0.24 ^g)^	0.19 ^g)^	‐	(0.70, 0.29)^g)^	[40]
TCPQ	30	718 ^g)^	5.4/2.5/‐^g)^	0.38 ^g)^	0.43 ^g)^	‐	(0.67, 0.30)^g)^	[41]
DCPPr‐α‐NDPA	42.7	748 ^g)^	1.9/1.7/‐^g)^	0.25 ^g)^	0.22 ^g)^	‐	(0.69, 0.30)^g)^	[42]
DCPPr‐β‐NDPA	28.2	734 ^g)^	1.4/1.1/‐^g)^	0.07 ^g)^	0.06 ^g)^	‐	(0.68, 0.28)^g)^	[42]
DCPPr‐TPA	50.5	716 ^g)^	1.4/1.2/‐^g)^	0.11 ^g)^	0.11 ^g)^	‐	(0.70, 0.29)^g)^	[42]
DCPPr‐DBPPA	35.6	748 ^g)^	1.0/0.5/‐^g)^	0.04 ^g)^	0.04 ^g)^	‐	(0.70, 0.28)^g)^	[42]
CNPP‐TPA	56.9	684	8.7/‐/‐	2.8	2.2	LT_90_ (0.5 mA) = 8.5	(0.68, 0.31)	[43]
T‐β‐IQD	3.2	682	10.7/3.1/2.0	2.7	‐	‐	(0.69, 0.31)	[44]
	4.5	711 ^g)^	9.4/‐/‐^g)^	0.6 ^g)^	‐	‐	(0.71, 0.29)^g)^	[44]
TIQD	2.7	677	8.7/3.1/2.0	2.7	‐	‐	(0.69, 0.31)	[44]
	3.6	709 ^g)^	6.6/‐/‐^g)^	0.64 ^g)^		‐	(0.71, 0.29)^g)^	[44]
TPA‐DQP	107	676	18.3/3.1/0.9	5.7	4.7	‐	(0.67, 0.32)	[45]
*p*TPA‐DPPZ	5.0	652 ^g)^	12.3/11.6/10.4 ^g)^	8.1 ^g)^	3.6 ^g)^	‐	(0.67, 0.33)^g)^	[46]
APPT‐BPXZ	1.4	665	2.0/‐/‐	0.8	0.5	‐	(0.67, 0.33)	[47]
*m*DPBPZ‐PXZ	‐	680 ^g)^	5.2/3.1/1.9 ^g)^	2.8 ^g)^	2.3 ^g)^	‐	(0.68, 0.32)^g)^	[48]
TPA‐PZCN	‐	680 ^g)^	5.3/4.5/‐^g)^	1.4 ^g)^	1.3 ^g)^	‐	(0.69, 0.30)^g)^	[49]
CN‐TPA	5.8	688	18.4/1.0/‐	5.0	5.8	LT_90_ (100 cd m^−2^) = 0.3	(0.68, 0.32)	[50]
TPA‐CN‐N4	11.0	688	21.2/9.2/‐	5.7	6.3	‐	(0.68, 0.32)	[51]
TPA‐CN‐N4‐2PY	15.8	702	22.8/5.1/‐	4.6	4.8	‐	(0.68, 0.32)	[51]
Py‐TPA	115	726	22.2/‐/‐	3.8	4.2	‐	(0.68, 0.32)	[52]
Py‐CN‐TPA	73.6	722	16.5/5.2/‐	3.6	4.1	‐	(0.69, 0.31)	[52]
TPACNBz	1.5	712	6.6/4.9/2.7	‐	‐	‐	(0.68, 0.29)	[53]
SDPA‐APDC	9.6	696	10.8/8.1/4.2	‐	‐	‐	(0.69, 0.30)	[54]
2PN	14.0	701	0.04/‐/‐	0.04	0.02	‐	(0.67, 0.33)	[55]

^a)^ Delayed lifetime in film state

^b)^ peak wavelength of EL spectrum

^c)^ maximum external quantum efficiency, external quantum efficiency at 100 cd m^−2^, and external quantum efficiency at 1000 cd m^−2^

^d)^ maximum current efficiency

^e)^ maximum power efficiency

^f)^ CIE coordinates of EL spectrum

^g)^ nondoped OLED.

**Table 2:** PL and EL data of MR‐type red TADF emitters discussed in Section 3.2

Emitter	PL data			EL data								Reference
	λ_PL_ ^a)^ [nm]	FWHM^b)^ [nm]	τ_D_ ^c)^ [µs]	Sensitizer	λ_EL_ ^d)^ [nm]	FWHM^e)^ [nm]	EQE_max/100/1000_ ^f)^ [%]	CE_max_ ^g)^ [cd A^−1^]	PE_max_ ^h)^ [lm W^−1^]	Lifetime [h]	CIE (x, y)^i)^	
BBCz‐R	615	21	‐	n/a	616	26	22.0/2.1/‐	‐	‐	‐	(0.67, 0.33)	[56]
R‐BN	662	38	‐	Ir(mphmq)_2_tmd	664^j)^	48^j)^	28.1/‐/‐^j)^	‐	‐	LT_90_ (2000 cd m^−2^) = 125^j)^	(0.719, 0.280)^j)^	[57]
R‐TBN	692	38	‐	Ir(mphmq)_2_tmd	686^j)^	49^j)^	27.6/‐/‐^j)^	‐	‐	LT_90_ (2000 cd m^−2^) = 151^j)^	(0.721, 0.278)^j)^	[57]
BNO3	616	33	‐	PO‐01	626^j)^	41^j)^	27.2/25.2/20.8^j)^	26.6^j)^	29.5^j)^	LT_90_ (1 mA) = 36.9^j)^	(0.67, 0.33)^j)^	[59]
BNNO	637	32	‐	Ir(piq)_2_acac	643^j)^	42^j)^	34.4/34.0/30.1^j)^	25.2^j)^	24.1^j)^	LT_95_ (5000 cd m^−2^) = 605^j)^	(0.708, 0.292)^j)^	[60]
BN‐R	624	34	71.8	n/a	627^k)^	48^k)^	17.5/6.1/2.2^k)^	16.9^k)^	16.1^k)^	LT_50_ (876 cd m^−2^) = 94.7^k)^	(0.681, 0.312)^k)^	[61]
PPZ‐BN	613	48	‐	PO‐01	613^j)^	55^j)^	26.9/26.8/26.0^j)^	34.5^j)^	34.6^j)^	LT_99_ (10 000 cd m^−2^) = 43^j)^	(0.66, 0.34)^j)^	[62]
*p*BDPA‐TOAT	603	48	308	n/a	624	62	11.3/3.1/1.1	12.6	11.7	‐	(0.66, 0.34)	[64]

^a)^ Peak wavelength of PL spectrum in dilute toluene solution

^b)^ full width at half maximum of PL spectrum

^c)^ delayed lifetime in film state

^d)^ peak wavelength of EL spectrum

^e)^ full width at half maximum of EL spectrum

^f)^ maximum external quantum efficiency, external quantum efficiency at 100 cd m^−2^, and external quantum efficiency at 1000 cd m^−2^

^g)^ maximum current efficiency;

^h)^ maximum power efficiency

^i)^ CIE coordinates for EL spectrum

^j)^ PSTADF OLED

^k)^ solution‐processed OLED.

### 3.1 Conventional D‐A‐Type Red TADF Emitters

**Figure 3:**
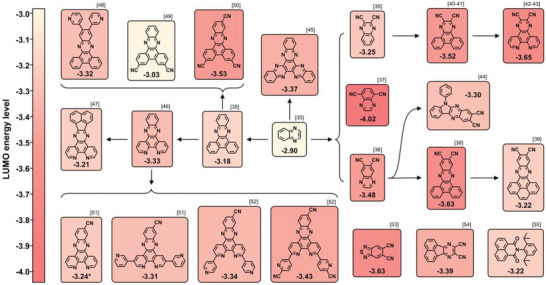
Derived routes of QNO‐based acceptors.

**Figure 4:**
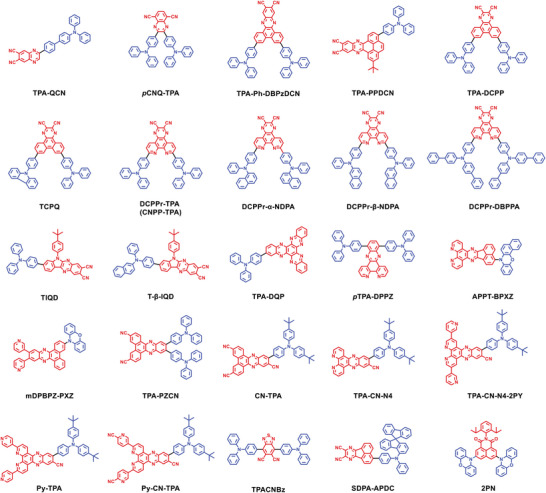
Molecular structures of D‐A‐type red TADF emitters discussed in Section 3.1.

A red/deep‐red emission corresponds to a small optical bandgap. This means that red/deep‐red TADF emitters generally suffer from more serious nonradiative decays induced by structural relaxations than blue and green ones, according to the energy gap law. A plausible approach is employing rigid molecular components to suppress unfavorable nonradiative loss.^[^ ^34^ ^]^ That is why the currently developed D‐A type emitters aiming at the BT.2020 red standard generally contain planar and rigid acceptors, which exhibit very deep LUMO energy levels. To obtain deeper red emissions, most studies have focused on the structural modifications of acceptor segments, which can reduce the energy gaps and thus redshift the emission bands. As depicted in **Figure** **3** and **Figure** **4**, quinoxaline (QNO) is an ideal prototype to derivate acceptors that can be used to construct D‐A‐type red emitters.^[^ ^35^ ^]^ By employing triphenylamine (TPA) as donor and quinoxaline‐6,7‐dicarbonitrile (QCN) as acceptor, Wang and coworkers reported a unique D‐π‐A type TADF emitter **TPA‐QCN**.^[^ ^36^ ^]^ Although **TPA‐QCN** only exhibits orange‒red emission in dilute toluene solution, intensive deep‐red emissions with peaks shifting from 649 to 700 nm were observed when **TPA‐QCN** was employed as a dopant in solid films. Similar phenomena are often noticed because intermolecular packing can result in emission redshifts for D‐A‐type TADF emitters, especially for emitters with a relatively long conjugated framework. Moreover, benefiting from the edge‐to‐edge arrangement based on multi‐intermolecular interactions of **TPA‐QCN**, the highly doped and neat films also maintained decent photoluminescence quantum yields (PLQYs) of over 20%. Consequently, the doped OLED based on **TPA‐QCN** achieves an EQE_max_ as high as 9.4% with an EL peak (λ_EL_) at 700 nm and CIE coordinates of (0.68, 0.31). Furthermore, its nondoped OLED exhibits near‐infrared EL with an EQE_max_ of 3.9%, λ_EL_ at 728 nm and CIE coordinates of (0.69, 0.31). Subsequently, by changing the substitution position of cyano groups on QCN, Xu and coworkers developed an isomeric acceptor quinoxaline‐5,8‐dicarbonitrile (*p*CNQ) and thus designed a deep‐red TADF emitter ***p*CNQ‐TPA**.^[^ ^37^ ^]^ ***p*CNQ‐TPA** features a quasi‐planar configuration, where the integrant groups *p*CNQ and TPA are well regulated to manipulate molecular orientation and intermolecular interactions. In the doped film, ***p*CNQ‐TPA** exhibits deep‐red emission with a peak at 691 nm, a horizontal ratio of 74.5%, and a PLQY of 90%. The OLED employing ***p*CNQ‐TPA** as the emitter achieves an extremely high EQE_max_ of 30.3% with λ_EL_ at 660 nm and CIE coordinates of (0.69, 0.31). These results indicate that peripheral cyano modification is an effective approach to enhance the electron‐withdrawing ability of acceptor units and achieve deep‐red emission.

To further enhance the electron‐accepting abilities of such units, Wang and coworkers developed a large‐size acceptor dibenzo[*a*,*c*]‐phenazine‐11,12‐dicarbonitrile (DBPzDCN), by extending the π‐conjugated length.^[^ ^38^ ^]^ By connecting the DBPzDCN core with two DPA groups, a new TADF emitter **DPA‐Ph‐DBPzDCN** was thus developed, showing red emission with a peak at 618 nm in toluene. Benefiting from the strong long‐range CT feature, the corresponding OLED exhibits deep‐red EL peaking at 708 nm, with CIE coordinates of (0.69, 0.30). However, the device shows an unsatisfactory EQE of only 5.5%. To improve the OLED performance, the same group reported a new TADF emitter **TPA‐PPDCN** by further extending the π‐conjugated framework of DBPzDCN.^[^ ^39^ ^]^ The large π‐conjugated acceptor endows **TPA‐PPDCN** with red to deep‐red emission and high PLQYs above 70% in doped films at various doping concentrations. As a result, the **TPA‐PPDCN**‐based OLED shows deep‐red EL with CIE coordinates of (0.70, 0.30), which is close to the BT.2020 red standard. Moreover, the **TPA‐PPDCN**‐based OLED also achieves a remarkable EQE_max_ of 16.4%.

By employing a similar modification on the QCN‐isomeric acceptor unit, Wang and coworkers further reported a deep‐red/near‐infrared TADF emitter **TPA‐DCPP**.^[^ ^40^ ^]^ Owing to the aggregation‐induced emission (AIE) characteristic, **TPA‐DCPP** exhibits deep‐red emission peaking at 708 nm with a high PLQY of 14% in the neat film. Consequently, the nondoped OLED employing **TPA‐DCPP** as the emitter achieves an EQE_max_ of 2.1% with deep‐red CIE coordinates of (0.70, 0.29). Notably, the doped OLED shows a higher EQE of 9.8% with λ_EL_ at 668 nm and CIE coordinates of (0.68, 0.32). Following this report, Chi and coworkers proposed a bilateral asymmetric strategy and developed a D‐A‐D′ type TADF emitter **TCPQ**.^[^ ^41^ ^]^ Asymmetric substitution with two different donors breaks the molecular symmetry of **TCPQ**, resulting in a high radiative rate (*k*_r_) of 1.7×10^7^ s^−1^ and a decent reverse intersystem crossing (RISC) rate (*k*_RISC_) of 2×10^4^ s^−1^. Moreover, the coexistence of multiple donors endows **TCPQ** with dep‐red emission peaking at 709 nm in the neat film. The nondoped OLED achieves an improved EQE_max_ of 5.4% with deep‐red EL peaking at 718 nm and CIE coordinates of (0.70, 0.30).

In addition to expanding the π‐conjugated structure by pure hydrocarbon groups, heterocyclic extension is also a commonly used strategy. In 2021, Tang and coworkers reported a series of tailor‐made red TADF emitters, **DCCPr‐*α*‐NDPA**, **DCCPr‐*β*‐NDPA**, **DCPPr‐TPA**, and **DCCPr‐DBPPA**, which comprise the same acceptor pyridine‐conjugated pyrazino[2,3‐f][1,10]phenanthroline‐2,3‐dicarbonitrile (DCCPr) and various electron‐donating DPA derivatives.^[^ ^42^ ^]^ Owing to the planar and rigid DCPPr core, these emitters can form intramolecular hydrogen bonds, which are conducive to improving the emissive efficiency and promoting horizontal orientation and showing deep‐red emissions (692–710 nm). As a result, nondoped OLEDs based on **DCCPr‐α‐NDPA**, **DCCPr‐β‐NDPA**, **DCPPr‐TPA**, and **DCCPr‐DBPPA** showed EL performances with EQE_max_s of 1.9%, 1.4%, 1.4%, and 1.0%, λ_EL_s at 716, 748, 734, and 748 nm, and CIE coordinates of (0.69, 0.30), (0.68, 0.28), (0.70, 0.29), and (0.70, 0.28), respectively. An emitter with the same molecular structure as **DCPPr‐TPA** was also reported by Xu and coworkers, named **CNPP‐TPA**.^[^ ^43^ ^]^ To suppress the enhanced concentration quenching induced by π‐π stacking of extended acceptors, Ge and coworkers proposed a strong fused ring acceptor and developed two deep‐red TADF emitters, **T‐*β*‐IQD** and **TIQD**.^[^ ^44^ ^]^ The unique molecular structures give **T‐*β*‐IQD** and **TIQD** several advantages, such as efficient AIE characteristics, J‐aggregate packing modes, and large face‐to‐face distances in crystalline states. With these features, **T‐*β*‐IQD** and **TIQD** both show concentration insensitivity, indicating well‐suppressed concentration quenching induced by intermolecular π‐π stacking, which eventually drastically improves the emissive efficiencies in the neat films. Consequently, the **T‐*β*‐IQD** and **TIQD** based nondoped OLEDs exhibit high‐efficiency deep‐red EL with CIE coordinates of both (0.71, 0.29) and state‐of‐the‐art EQE_max_s of 9.4% and 6.6%, respectively.

Aside from cyano‐containing QNO derivatives, some cyano‐free QNO derivatives with large π‐conjugated frameworks and strong electron‐accepting abilities are also popular in the molecular design of red/deep‐red TADF emitters. In 2020, Wang and coworkers developed a novel multifunctional emitter **TPA‐DQP** by employing a large π‐conjugated (diquinoxalino[2,3‐a:2′,3′‐c]phenazine) DQP as the acceptor.^[^ ^45^ ^]^ The DQP framework, fused by three QNO units, has a large π‐conjugated planar framework, which allows diverse packing modes in crystalline states. In the solid state, **TPA‐DQP** not only shows interesting mechanochromic luminescence behavior but also demonstrates efficient TADF characteristics induced by molecular packing. The OLED using **TPA‐DQP** as the emitter presents a decent EQE_max_ of 18.3% and shows deep‐red emission with CIE coordinates of (0.67, 0.32). Subsequently, Xu and coworkers reported a novel deep‐red TADF emitter ***p*TPA‐DPPZ** featuring the T‐shape molecular structure as two TPA donors substituted at both sides of the dipyridophenazine (DPPZ) acceptor.^[^ ^46^ ^]^ The rational spatial arrangement of the functional groups leads to limited but sufficient molecular packing for the enhanced RISC process, giving rise to a high PLQY of 87% in its neat film. In addition, the neat film also shows improved carrier transport ability. By employing a nondoped bilayer OLED structure, ***p*TPA‐DPPZ** realizes a state‐of‐the‐art EQE_max_ of 12.3%, accompanied by deep‐red EL with a peak at 652 nm and CIE coordinates of (0.67, 0.33). Following this report, Yang and coworkers developed a new acceptor acenaphtho [1′,2′:5,6]pyrazino [2,3‐f] [1,10] phenanthroline (APPT), and connected it to a strong donor 7H‐benzo [c]phenoxazine (BPXZ), to give a red emitter, **APPT‐BPXZ**. Benefiting from the highly torsional configuration, **APPT‐BPXZ** exhibits a rather small ΔE_ST_ value of 0.04 eV, which provides a *k*_RISC_ up to 10^6^ s^−1^. The red OLED employing **APPT‐BPXZ** as emitter achieves an EQE_max_ of 2.0% with λ_EL_ at 665 nm and CIE coordinates of (0.67, 0.33).^[^ ^47^ ^]^

To further develop deep‐red TADF emitters, peripheral modifications of the above large π‐conjugated QNO derivatives were also developed in the corresponding literature. By substituting two flexible pyridine groups, Zhang and coworkers reported a red emitter **mDPBPZ‐PXZ** that can be used to construct deep‐red nondoped OLEDs.^[^ ^48^ ^]^ In its crystal, two released pyridine groups play a significant role in providing suitable steric hindrance against intermolecular π‐π stacking, which is expected to suppress concentration quenching, thus resulting in a superior emissive efficiency with a high PLQY of 33% in the neat film. The nondoped OLED based on **mDPBPZ‐PXZ** exhibits deep‐red EL with peak at 680 nm, CIE coordinates of (0.68, 0.32), and a high EQE_max_ of 5.2%. In the same year, Liao and coworkers reported a red emitter **TPA‐PZCN** by incorporating TPA as a donor and cyano‐modified dibenzo[a,c]phenazine‐3,6‐dicarbonitrile (PZCN) as an acceptor.^[^ ^49^ ^]^ As mentioned above, the introduction of the cyano group on the acceptor will significantly increase the electron‐accepting strength, leading to redshifted emission. Furthermore, due to the rigid planar structure and large steric hindrance between the donor and acceptor, the nondoped OLED based on **TPA‐PZCN** achieves a high EQE_max_ of 5.3%, and exhibits deep‐red EL with peak at 680 nm and CIE coordinates of (0.69, 0.30). As a follow‐up, Fan and coworkers reported a series of red to deep‐red emitters with pyridine or cyano or both substituted acceptors, and high‐performance deep‐red OLEDs with CIE coordinates closer to the BT.2020 red standard were further achieved.^[^ ^50^, ^51^, ^52^ ^]^

In addition to red/deep‐red emitters with QNO derivatives as acceptors, a handful of red/deep‐red emitters with other rigid acceptors have been reported. In 2020, Promarak and coworkers reported a deep‐red emitter **TPACNBz** with a simple and strong electron‐accepting 5,6‐dicyano[2,1,3] benzothiadiazole (CNBz) as acceptor.^[^ ^53^ ^]^ Two DPA groups substituted at the 4,7‐positions of CNBz form an effective D‐A‐D structure, giving rise to deep‐red emission with a relatively low ΔE_ST_ of 0.06 eV. The OLED using **TPACNBz** as the emitter achieves high EL performance with an EQE_max_ of 6.6%, λ_EL_ at 712 nm, and CIE coordinates of (0.68, 0.29). In 2021, Liao and coworkers reported a deep‐red TADF emitter **SDPA‐APDC** by incorporating a spiro‐type electron‐donating N,N‐diphenyl‐9,9′ ‐spirobi[fluorene]−2‐amine moiety (SDPA) into an electron‐accepting acenaphtho[1,2‐b]pyrazine‐8,9‐dicarbonitrile (APDC) unit.^[^ ^54^ ^]^ Due to the large steric hindrance of the SDPA donor, the intermolecular π‐π packing of **SDPA‐APDC** is well suppressed, which allows a high PLQY of 80.5% in the neat film. Consequently, the **SDPA‐APDC**‐based OLED achieves a high EQE_max_ of 10.8% with deep‐red EL peaking at 696 nm and CIE coordinates of (0.69, 0.30). In 2022, Xie and coworkers reported a series of 1,8‐Naphthalimide‐based wedge‐shape emitters by adopting multiple donor units and isopropyl modification strategies.^[^ ^55^ ^]^ Among these emitters, **2PN** exhibits deep‐red EL with peak at 701 nm and CIE coordinates of (0.67, 0.33).

### 3.2 MR‐Type Red TADF Emitters

**Figure 5:**
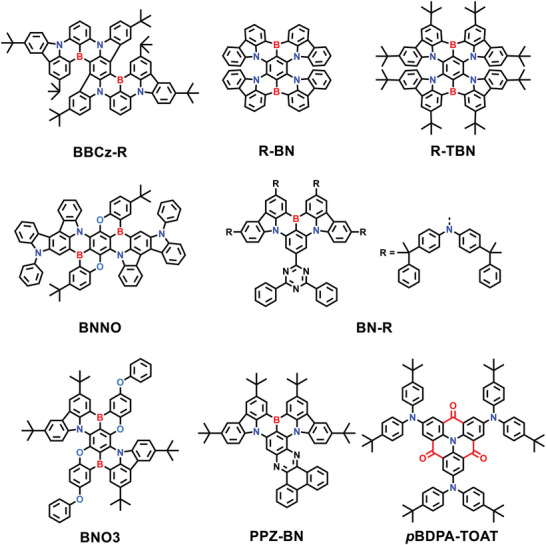
Molecular structures of MR‐type red TADF emitters discussed in Section 3.2.

Since the pioneering work in 2016 by Hatakeyama and coworkers, numerous MR‐type TADF emitters that can cover the full‐color visible range have been actively developed in recent years.^[^ ^21^, ^23^, ^24^, ^25^ ^]^ Although MR‐type emitters are theoretically superior to D‐A‐type emitters in approaching the BT.2020 red standard, only a few eligible red MR‐type emitters have been reported. This is mainly because the developed MR skeletons emit in the blue‐to‐green region, and molecular designs for a large spectral shift are very challenging. To approach redshifted emissions toward the BT.2020 red standard, current efforts are focusing on enhancing the CT features inside MR‐type emitters. This can be achieved by either constructing MR skeletons with B‐π‐B/N‐π‐N *para*‐disposed patterns or introducing electron‐donating/accepting peripheral substitutions onto MR skeletons (**Figure** **5**).

The first pure red MR‐type emitter was reported by Yasuda and coworkers in 2020.^[^ ^56^ ^]^ In this report, they proposed an epoch‐making design strategy for exploring full‐color MR‐type TADF emitters from deep‐blue to deep‐red. In particular, they first proposed that well‐arranged *para* B‐π‐B and N‐π‐N patterns in the MR polycyclic framework can significantly increase the acceptor and donor strengths, resulting in an evidently reduced energy gap and thus facilitating redshifted emission. The proof‐of‐concept emitter **BBCz‐R** shows pure‐red emission peaking at 615 nm with an extremely small FWHM of 21 nm in dilute toluene solution. The OLED employing **BBCz‐R** as the emitter exhibits pure‐red EL with an EQE_max_ of 22.0%, λ_EL_ of 616 nm, FWHM of 26 nm and CIE coordinates of (0.67, 0.33). Using similar *para* B‐π‐B and N‐π‐N patterns, Duan and coworkers developed two deep‐red/near‐infrared MR‐type emitters **R‐BN** and **R‐TBN**.^[^ ^57^ ^]^ Around a central phenyl ring, there are three pairs of *para* B/N atoms in **R‐BN** and **R‐TBN**, which significantly reduce the energy gaps and thus give rise to deep‐red emissions peaking at 629 and 651 nm with FWHMs of both 38 nm in dilute toluene solution, representing the longest‐wavelength MR‐type emitters ever reported. The corresponding OLEDs based on the phosphor‐sensitized TADF (PSTADF) system exhibit record‐red EL with peaks at 664 and 686 nm and CIE coordinates of (0.719, 0.280) and (0.721, 0.278) for **R‐BN** and **R‐TBN**, respectively. It is noteworthy that such CIE coordinates far exceed the BT.2020 red standard, which outperforms all reported values of red/deep‐red/near‐infrared TADF emitters. Moreover, these OLEDs also achieve outstanding EQE_max_s of 28.1% and 27.6%, respectively. At the same time, Wang and coworkers demonstrated the excellent chiral properties of such MR skeleton, thereby providing an appealing avenue to the future exploitation of high‐performance chiroptical materials.^[^ ^58^ ^]^

By further incorporating oxygen (O) atoms, Yang and coworkers proposed a *para*‐B/N/O patterned framework for red MR‐type emitters.^[^ ^59^ ^]^ Similar to the abovementioned *para*‐position patterns, the *para* N‐π‐N, O‐π‐O, and B‐π‐B pattern in **BNO1‐3** could also enhance the CT effect by increasing the electron‐donating and electron‐accepting strengths, thereby leading to redshifted emissions. Nonetheless, due to the weaker electron‐donating ability of O atoms than N atoms, **BNO1‐3** exhibit emissive peaks ranging from 605–616 nm in dilute toluene solution, which are obviously blueshifted compared to that of the aforementioned **R‐BN**. Only the **BNO‐3**‐based OLED achieves pure‐red EL that approaches the BT.2020 red standard, with a peak at 626 nm, FWHM of 41 nm and CIE coordinates of (0.67, 0.33). Most recently, Duan and coworkers made further structural modifications to **BNO1** by embedding 5‐phenyl‐5,8‐dihydroindolo[2,3‐c]carbazole (23cICz) as multiple nitrogen sources.^[^ ^60^ ^]^ The N‐π‐N pattern in 23cICz contributes to enhancing the electron‐donating ability, whereas the large rigid planar structure of 23cICz greatly expands the π‐conjugated framework of the MR skeleton to suppress the molecular geometry change and vibrational relaxation. Consequently, the obtained pure‐red emitter **BNNO** shows pure‐red emission with a peak at 637 nm and a small FWHM of only 32 nm in dilute toluene solution. The PSTADF‐OLEDs based on **BNNO** obtained CIE coordinates of (0.708, 0.292), which is in line with the BT.2020 red standard. Moreover, benefiting from the preferential horizontal orientation of **BNNO** and efficient energy transfer process from the phosphorescent sensitizer to **BNNO**, this device also exhibits a considerably high EQE_max_ of 34.4%, along with an extremely long operational lifetime (LT_95_) of 605 h at an initial brightness of 5000 cd m^−2^, representing the best comprehensive OLED performance of red TADF emitters reported thus far.

In addition to the adjustment of atomic arrangement patterns, strategies of introducing peripheral electron‐donating and electron‐accepting substitutions are also feasible for redshifted emission. In 2022, Wang and coworkers proposed a synergistic strategy to construct red MR‐type emitters by simultaneously introducing auxiliary electron donor and acceptor moieties.^[^ ^61^ ^]^ The proof‐of‐concept emitter **BN‐R** that exhibits narrowband pure‐red emission with a peak at 624 nm and an FWHM of 46 nm in dilute toluene solution was thereby developed. The solution‐processed OLED with **BN‐R** realizes outstanding EL performance with a decent EQE_max_ of 17.5% and pure‐red CIE coordinates of (0.681, 0.312). Most recently, Duan and coworkers reported a pure‐red MR‐type emitter **PPZ‐BN** by fusing MR skeletons with an electron‐accepting moiety phenanthro[9,10‐b]pyrazine (PPZ).^[^ ^62^ ^]^ The introduction of PPZ not only greatly enhances the degree of π‐conjugation and CT effect, but also maintains the fundamental MR feature, leading to a significant redshifted narrowband emission of **PPZ‐BN**. As a result, **PPZ‐BN** displays vivid pure‐red emission peaking at 613 nm with a small FWHM of 48 nm in dilute toluene solution. The corresponding PSTADF‐OLED shows pure‐red EL with λ_EL_ at 613 nm, FWHM of 55 nm, CIE coordinates of (0.66, 0.34), and an EQE_max_ of 26.9%.

In addition to the B/N‐based MR system, the N/carbonyl system presents an alternative option capable of inducing MR characteristics and can therefore be employed in constructing red MR‐type emitters.^[^ ^63^ ^]^ To clarify the importance of excited state alignments of MR‐based D‐A emitters in determining their preferring characteristics, Zhang and coworkers reported a series of red MR‐type emitters with D_3_‐A structures.^[^ ^64^ ^]^ These emitters, which possess the same MR‐type skeleton as the acceptor unit, exhibit differentiated photophysical properties in terms of FWHMs and TADF characteristics resulting from competitive contributions of MR and intermolecular CT. As the most superior among them, ***p*BDPA‐TOAT** exhibits red emission with a peak at 603 nm and an FWHM of 48 nm in dilute toluene solution. The OLED based on ***p*BDPA‐TOAT** exhibits deep‐red EL with λ_EL_ at 613 nm, an FWHM of 55 nm, CIE coordinates of (0.66, 0.34), and EQE_max_ of 26.9%. This work provides insight into the trade‐off between the spectral redshift and broadening of MR‐type emitters when introducing D‐A‐type structures, which could facilitate the development of MR‐type emitters that approach longer wavelengths.

## 4 TADF Emitters toward the BT.2020 Green Standard

**Figure 6:**
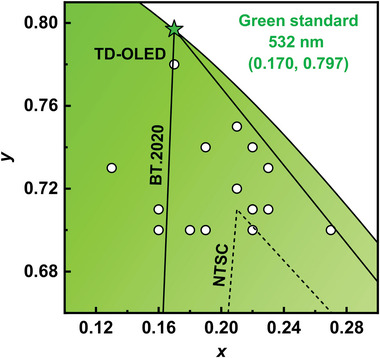
The CIE coordinates of all the summarized MR‐type TADF emitters in the green region.

**Figure 7:**
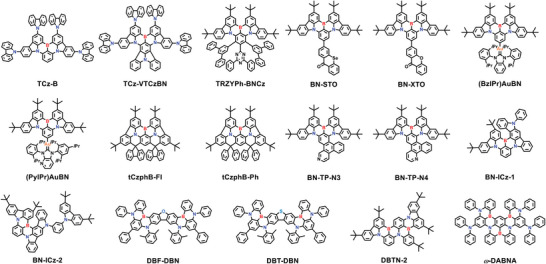
Molecular structure of MR‐type green TADF emitters discussed in Section 4.

The green band falls within the central section of the whole visible spectrum, and the corresponding valid range of green is noticeably narrower than the blue and red bands. The BT.2020 green standard mandates ultrahigh spectral quality, necessitating both appropriate wavelength peaks and narrow spectral widths, posing a significant challenge for TADF emitters to achieve CIE coordinates that approach the BT.2020 green standard, as even a slight shift in peak wavelength or spectral width broadening can result in coverage of adjacent blue or red regions and thus harm the overall color quality. It is exceedingly challenging for D‐A‐type TADF emitters to effectively shift CIE coordinates close to the BT.2020 green standard due to their broad emission characteristics. To the best of our knowledge, the CIE_y_ record of conventional D‐A‐type TADF emitters is only 0.64, which is far from meeting the NTSC green standard.^[^ ^65^ ^]^ By contrast, MR‐type TADF emitters can easily meet the required narrow spectral widths; however, it remains a great challenge to achieve appropriate peak wavelengths and spectral profiles. To date, only certain green MR‐type emitters possess the potential to attain CIE coordinates that approach the BT.2020 green standard, as outlined in this section (**Figure** **6** and **Figure** **7**, **Table** **3**).

**Table 3:** PL and EL data of MR‐type green TADF emitters discussed in Section 4

Emitter	PL data			EL data								Reference
	λ_PL_ ^a)^ [nm]	FWHM^b)^ [nm]	τ_D_ ^c)^ [µs]	Sensitizer	λ_EL_ ^d)^ [nm]	FWHM^e)^ [nm]	EQE_max/100/1000_ ^f)^ [%]	CE_max_ ^g)^ [cd A^−1^]	PE_max_ ^h)^ [lm W^−1^]	Lifetime [h]	CIE (x, y)^i)^	
Tcz‐B	512	27	71	n/a	515	30	29.2/24.7/9.4	100.7	72.4	‐	(0.16, 0.71)	[66]
TCz‐VTCzBN	521	29	9.9	n/a	524	37	32.2/17.8/15.6	129.3	96.7	‐	(0.22, 0.71)	[67]
TRZCzPh‐BNCz	513	29	8.7	n/a	513	33	31.4/29.5/23.1	‐	84.1	‐	(0.16, 0.70)	[68]
BN‐STO	517	34	25.3	n/a	517	34	40.1/39.0/28.1	141.2	176.9	LT_50_ (1000 cd m^−2^) = 35	(0.19, 0.70)	[72]
BN‐XTO	515	33	57.1	n/a	513	34	37.3/34.1/18.6	131.0	156.9	LT_50_ (1000 cd m^−2^) = 354	(0.19, 0.70)	[72]
(BzIPr)AuBN	511^j)^	30^j)^	5.5	n/a	513	34	30.3/30.0/28.1	82.7	‐	LT_50_ (1000 cd m^−2^) = 1210	(0.18, 0.70)	[73]
(PyIPr)AuBN	511^j)^	30^j)^	5.9	n/a	517	38	27.3/26.5/19.7	96.1	‐	‐	(0.19, 0.70)	[73]
BN‐ICz‐1	521	20	‐	3CTF	523^k)^	23^k)^	30.5/29.6/26.1^k)^	‐	84.2^k)^	LT_90_ (2000 cd m^−2^) = 82.1^k)^	(0.22, 0.74)^k)^	[75]
			‐	Ir(ppy)_2_acac	525^l)^	19^l)^	‐	220^l)^	‐		(0.17, 0.78)^l)^	[75]
BN‐ICz‐2	520	21	‐	3CTF	523^m)^	23^m)^	29.8/26.7/15.6^m)^	‐	102.9^j)^	LT_90_ (2000 cd m^−2^) = 71.3^m)^	(0.23, 0.73)^m)^	[75]
BN‐TP‐N3	519	32	55.4	n/a	524	33	37.3/34.7/19.8	135.3	139.9	LT_90_ (32 400 cd m^−2^) = 34.0^k)^	(0.23, 0.71)	[76]
BN‐TP‐N4	520	32	49.5	n/a	528	35	36.5/34.1/21.2	136.3	143.2	‐	(0.27, 0.70)	[76]
DBF‐DBN	514	22	39	n/a	521	30	21.5/2.9/0.9	82.4	76.2	‐	(0.22, 0.71)	[77]
DBT‐DBN	516	19	33	n/a	520	24	31.3/14.8/4.1	116.2	93.6	‐	(0.22, 0.70)	[77]
tCzphB‐Ph	523	21	372	BCz‐o‐TRZ	527^m)^	24^m)^	29.3/20.4/9.1^m)^	‐	‐	LT_90_ (21 400 cd m^−2^) = 1.4^m)^	(0.21, 0.75)^m)^	[78]
tCzphB‐Fl	532	21	412	BCz‐o‐TRZ	535^m)^	26^m)^	26.2/20.0/8.1^m)^	‐	‐	LT_90_ (24 000 cd m^−2^) = 70.5^m)^	(0.21, 0.72)^m)^	[78]
ω‐DABNA	509	18	9.0	n/a	512	25	31.1/30.8/29.4	101.0	98.1	LT_80_ (1000 cd m^−2^) = 292	(0.13, 0.73)	[79]
DBTN‐2	512	20	6.5	n/a	520	29	35.2/33.6/20.4	132.9	130.4	‐	(0.19, 0.74)	[80]

^a)^ Peak wavelength of PL spectrum in dilute toluene solution

^b)^ full width at half maximum of PL spectrum

^c)^ delayed lifetime in film state

^d)^ peak wavelength of EL spectrum

^e)^ full width at half maximum of EL spectrum

^f)^ maximum external quantum efficiency, external quantum efficiency at 100 cd m^−2^, and external quantum efficiency at 1000 cd m^−2^;

^g)^ maximum current efficiency

^h)^ maximum power efficiency

^i)^ CIE coordinates for EL spectrum

^j)^ in dilute tetrahydrofuran solution

^k)^ PSTADF OLED

^l)^ top‐emitting OLED

^m)^ HF(TADF‐sensitized) OLED.

Modifying MR skeletons that are already close to the ideal emitting region is a facile mean for precisely modulating emission positions, while it may induce more pronounced structural relaxations and thus broader emission bandwidths, i.e., a trade‐off between spectral shifting and broadening. A rigid MR precursor is thus highly desired to suppress spectral broadening during modifications, and versatile boron‐embedded 1,3‐bis(carbazol‐9‐yl)benzene CzBN is an ideal candidate. For this purpose, in 2021 Yasuda and coworkers demonstrated an easy‐to‐use and straightforward molecular engineering approach by incorporating electron‐accepting imine and electron‐donating amine moieties on CzBN, which enables systematic hypsochromic and bathochromic shifts of narrowband emissions.^[^ ^66^ ^]^ The peripheral carbazole (Cz) moieties successfully increase the electron‐donating ability of CzBN, leading to a distinct decrease in the bandgap. As a result, the green emitter **TCz‐B** exhibits narrowband green emission peaking at 512 nm with an FWHM of 27 nm in dilute toluene solution, along with an extremely high PLQY of ≈100%. The corresponding narrowband green OLED demonstrates a reasonably high EQE_max_ of 29.2% with λ_EL_ at 515 nm, FWHM of 30 nm and CIE coordinates of (0.16, 0.71). Notably, the CIE_y_ of this device is in line with the NTSC green standard, representing a breakthrough in green OLEDs based on TADF emitters. Following this report, Zheng and coworkers derived a pure‐green MR‐type emitter **TCz‐VTCzBN** based on the parent **TCz‐B**.^[^ ^67^ ^]^ The incorporation of the indolo[3,2,1‐jk]carbazole unit allows **TCz‐VTCzBN** to show pure‐green emission with a peak at 521 nm and an FWHM of 29 nm, which is slightly greener than the parent **TCz‐B**. Moreover, due to its dual‐component fused hybridization of the MR effect, **TCz‐VTCzBN** delivers a small ΔE_ST_ value below 0.01 eV and a high PLQY of 98%. Consequently, the **TCz‐VTCzBN**‐based OLED exhibits standard pure‐green EL with λ_EL_ at 524 nm, FWHM of 37 nm and an improved EQE_max_ of 32.2%. It is noteworthy that the CIE coordinates of (0.22, 0.71) are decently consistent with the NTSC green standard.

MR‐type emitters generally show inefficient RISC processes, which lead to unsatisfactory efficiency roll‐offs in their corresponding OLEDs. In 2022, You and coworkers proposed a “space‐confined donor‐acceptor (SCDA)” strategy to accelerate the RISC process of MR‐type emitters.^[^ ^68^ ^]^ By inducing multiple donors and acceptors on the CzBN skeleton, two efficient pure‐green emitters **TRZCzPh‐BNCz** and **TRZTPh‐BNCz** were developed. The incorporation of SCDA units can enhance intermolecular CT characteristics, resulting in redshifted emissions; the steric hindrance between SCDA units can suppress spectral broadening induced by vibrational rotation, maintaining narrowband characteristics. In dilute toluene solution, **TRZCzPh‐BNCz** and **TRZTPh‐BNCz** display similar narrowband green emissions with peaks at 514 and 513 nm, and FWHMs of 34 and 29 nm, respectively. Furthermore, peripheral SCDA units also have the potential to form intermediate triplet states that are close to the S_1_ state, which may provide additional pathways for the RISC process, thus giving rise to *k*_RISC_s of approximately 10^6^ s^−1^. Consequently, OLEDs utilizing **TRZCzPh‐BNCz** and **TRZTPh‐BNCz** as emitters exhibit pure‐green narrowband EL with λ_EL_s both at 513 nm, FWHMs of 37 and 33 nm, respectively, and operate EQE_max_s of over 30% and decent efficiency roll‐offs. Although both devices show an identical peak wavelength, the slightly narrower EL spectrum of **TRZTPh‐BNCz** allows for a CIE_y_ value of up to 0.7, whereas **TRZCzPh‐BNCz** can only reach 0.68, demonstrating that spectral widths play a significant role in determining CIE coordinates in the green region.

Earlier studies have demonstrated that the incorporation of heavy atoms into the MR framework can enhance spin‐orbit coupling (SOC), thereby significantly promoting the RISC process.^[^ ^69^, ^70^, ^71^ ^]^ By introducing a peripheral group selenoxanthone containing heavy‐atom selenium (Se) onto the parent MR skeleton BNCz, Yang and coworkers reported a pure‐green MR‐TADF emitter **BN‐STO** and its O‐substituted counterpart **BN‐XTO**.^[^ ^72^ ^]^ The electron‐accepting selenoxanthone and xanthone groups at the para‐position of the B atom could narrow the energy gap, generating narrowband green emissions peaking at 506 and 502 nm with narrow FWHMs of 29 and 26 nm for **BN‐STO** and **BN‐XTO** in dilute toluene solution, respectively. Nevertheless, benefiting from the heavy atom effect induced by the Se atom, **BN‐STO** shows larger SOC and *k*_RISC_ values than **BN‐XTO**. Consequently, both TADF‐sensitized OLEDs display similar pure‐green EL with identical FWHMs of 34 nm and CIE coordinates of (0.19, 0.70) while differing in their efficiency performances. Specifically, the **BN‐XTO**‐based device achieves a high EQE_max_ of 37.3% with an EQE of 18.6% at 1000 cd m^−2^; the **BN‐STO**‐based device demonstrates better efficiency performance with an EQE_max_ of 40.1% and an EQE of 28.1% at 1000 cd m^−2^, indicating the superiority of the peripheral modification via heavy‐atom‐containing units.

Based on a similar design concept, both Che et al. and Yang et al. in 2022 described a simple gold (Au) coordination strategy to enhance the SOC and increase the *k*_RISC_ of MR‐type TADF emitters. A novel class of heavy metal Au(I)‐based green MR‐type TADF emitters was thus developed.^[^ ^73^, ^74^ ^]^ Theoretical calculations suggest that the 5d orbitals of Au atoms play a crucial role in enhancing both the SOC effect and *k*_RISC_, as they are involved in the lowest‐lying excited state. This permits Au(I) MR‐TADF emitters to have ultrafast *k*_RISC_ rates exceeding 10^7^ s^−1^, significantly surpassing those of the parent CzBN skeleton. Meanwhile, color purities of the Au(I) MR‐TADF emitters are only slightly affected by peripheral Au coordination due to the unchanged distribution of FMO distribution and structural rigidity. As a result, the TADF‐sensitized ultrapure green OLEDs based on **(BzIPr)AuBN** and **(PyIPr)AuBN** attained high CIE_y_ coordinates of up to 0.70 and outstanding EL performance with EQE_max_s reaching 30.3%, minimized roll‐offs down to 0.8% at 1000 cd m^−2^, high luminance exceeding 2.53×10^5^ cd m^−2^ and long operational lifetimes (LT_60_) of nearly 1210 h at an initial brightness of 1000 cd m^−2^.

To further suppress spectral broadening the side effect caused by peripheral modification, Duan and coworkers demonstrated a polycyclic aromatic hydrocarbon (PAH)‐fusion strategy for achieving ultrapure narrowband green emissions.^[^ ^75^ ^]^ Through precise synthetic fusion of the parent skeleton BNCz with 5,11‐diphenyl‐5,11‐dihydroindolo[3,2‐b]carbazole (ICz), extended π‐conjugated frameworks, enhanced structural rigidity, and suppressed vibration frequency could be simultaneously realized. The proof‐of‐concept emitters **BN‐ICz‐1** and **BN‐ICz‐2** exhibit ultra‐pure green emissions with dominant peaks at 521 and 520 nm and FWHMs of 20 and 21 nm in dilute toluene solution. The corresponding PSTADF‐OLEDs demonstrate high EL performance with considerably high EQE_max_s of 30.5% and 29.8% and favorable CIE coordinates of (0.22, 0.74) and (0.23, 0.73 for **BN‐ICz‐1** and **BN‐ICz‐2**, respectively. Furthermore, by adopting a top‐emitting OLED structure with a microcavity effect, **BN‐ICz‐1** delivers not only a record‐high current efficiency of 220 cd A^−1^ but also record‐pure green CIE coordinates of (0.17, 0.78), which are extremely close to the BT.2020 green standard, revealing the huge potential of MR‐type emitters in future ultra‐high‐resolution OLED displays.

Subsequently, Wang and coworkers demonstrated a unique nitrogen‐atom embedding molecular engineering (NEME) strategy to precisely regulate the peak wavelength of MR‐type emitters.^[^ ^76^ ^]^ The target emitters **BN‐TP‐N3** and **BN‐TP‐4** exhibit pure‐green emissions with peaks at 519 and 520 nm and FWHMs of both 32 nm in dilute toluene solution. The corresponding OLEDs exhibit pure‐green EL with λ_EL_s at 524 and 528 nm, FWHMs of 33 and 35 nm, and considerably high EQE_max_s of 37.3% and 36.5% for **BN‐TP‐N3** and **BN‐TP‐4**, respectively.

Most recently, Lan and coworkers reported dibenzo‐[*b*,*d*]furan‐ and dibenzo[*b*,*d*]thiophene‐fused pure‐green MR‐type emitters **DBF‐DBN** and **DBT‐DBN**.^[^ ^77^ ^]^ The double‐sized symmetric molecular structure allow **DBF‐DBN** and **DBT‐DBN** to show narrowband pure‐green emissions with peaks at 514 and 516 nm and small FWHMs of 22 and 19 nm, respectively. The OLEDs fabricated with **DBF‐DBN** and **DBT‐DBN** exhibit ultrapure EL with peaks at 521 and 520 nm, FWHMs of 31 and 24 nm and EQE_max_s of 21.5% and 31.3%, respectively. Moreover, it is noteworthy that the corresponding CIE coordinates of (0.22, 0.71) and (0.22, 0.70) of the two devices are also very close to the NTSC green standard.

To further boost the CIE coordinates of MR‐type emitters to approach the BT.2020 green standard, Zhang and coworkers proposed a multi‐lock strategy by locking the outer phenyl rings with the central phenyl ring in the parent skeleton BNCz using spiro‐carbon formation.^[^ ^78^ ^]^ Compared to peripheral modification, the multi‐lock strategy can endow MR‐type emitters with significantly suppressed excited‐state distortion and vibration modes caused by the strong intramolecular CT effect. In dilute toluene solution, the target emitters **tCzphB‐Ph** and **tCzphB‐Fl** show ultrapure green emissions with peaks at 523 and 531 nm and same FWHMs of 21 nm. They demonstrated the interesting solid‐state solvent effect, which has been previously overlooked, providing a satisfactory explanation for the broader spectra of **tCzphB‐Ph** and **tCzphB‐Fl** in doped film than in solution. Based on this understanding, they fabricated a **tCzphB‐Ph**‐based OLED, showing color‐saturated green EL with λ_EL_ at 527 nm, an FWHM of only 24 nm and CIE coordinates of (0.21, 0.75), representing the purest green emission reported for bottom‐emitting OLEDs ever reported; the **tCzphB‐Fl**‐based OLED also exhibits pure‐green EL with λ_EL_ at 535 nm, FWHM of 26 nm and CIE coordinates of (0.21, 0.72). Furthermore, by utilizing a commercial green phosphor Ir(ppy)_3_ as the sensitizer, a **tCzphB‐Ph**‐based PSTADF‐OLED demonstrates significantly improved efficiency roll‐off, maintaining an EQE of 30.6% even at an ultrahigh luminance over 1×10^5^ cd m^−2^.

Aside from the modification of the MR skeleton BNCz, the structural expansion of the well‐established blue MR‐type emitter **DABNA‐1** is also feasible to shift the CIE coordinates towards the BT.2020 green standard. In 2022, Hatakeyama and coworkers reported an ultrapure green MR‐emitter **ω‐DABNA**, which contains three B atoms and four N atoms. The target compound **ω‐DABNA** was synthesized by a new protocol—sequential multiple borylation—consisting of one‐pot borylation, amination, and one‐shot borylation.^[^ ^79^ ^]^ The expansion of the molecular framework not only narrows the energy gap, resulting in a redshifted narrowband emission, but also enhances the MR effect, allowing efficient TADF characteristics. Therefore, **ω‐DABNA** shows an ultranarrow green emission with a peak at 509 nm and a small FWHM of 18 nm in dilute toluene solution, along with a tiny ΔE_ST_ value of 11 meV in a doped PMMA film. The corresponding OLED based on **ω‐DABNA** exhibits pure‐green EL with λ_EL_ at 512 nm, an FWHM of 25 nm and CIE coordinates of (0.13, 0.73). In addition, the device achieves a high EQE_max_ of 31.1% with minimized efficiency roll‐off (EQEs of 29.4% at 1000 cd m^−2^ and 20.1% at 10 000 cd m^−2^). This work offers an innovative synthesis concept that can expand new synthetically accessible chemical space, which opens up new avenues for the development of multiple‐boron‐embedded MR‐TADF emitters.

Most recently, Zhang and coworkers reported an ultrapure green MR‐type emitter **DBTN‐2** via a highly distorted fused π‐conjugated molecular design concept.^[^ ^80^ ^]^ With this concept, on the one hand, the relaxation energy between the geometries of the excited and ground states is significantly reduced, leading to an ultrapure green narrowband at 512 nm with a small FWHM of only 20 nm; on the other hand, the different excitation characters of the singlet and triplet states (i.e., the ππ* excitation and the hybrid excitation of ππ* and πσ*, respectively) are induced, enhancing its SOC effect. Additionally, the introduction of multiple carbazole moieties gives rise to the charge‐resonance‐type excitation feature of the triplet states, thus resulting in a high density of triplet states. As a result, **DBTN‐2** exhibits a fast k_RISC_ of up to 10^5^ s^−1^ in the doped film. The corresponding OLED demonstrates ultrapure green EL with λ_EL_ at 520 nm, an FWHM of 29 nm and a considerably high EQE_max_ of 35.2% alongside suppressed efficiency roll‐off. Notably, the CIE coordinates of (0.18, 0.74) far exceed the NTSC green standard and approach the BT.2020 green standard, indicating its potential for improving the performance of current OLED displays. This work provides a novel design concept for more MR‐TADF emitters with high efficiencies and excellent color purities.

## 5 TADF Emitters toward the BT.2020 Blue Standard

**Figure 8:**
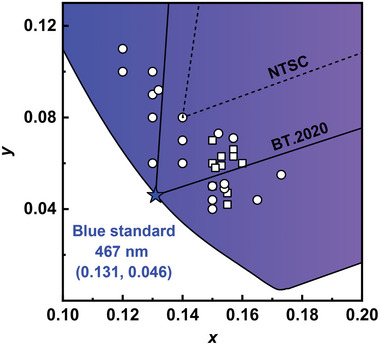
The CIE coordinates of all the summarized D‐A‐type (square) and MR‐type (circle) TADF emitters in the blue region.

The blue band occupies the far‐left end of the visible spectrum. The unique spectral position provides ample room for both D‐A‐type and MR‐type emitters to approach the BT.2020 blue standard, similar to red bands. According to the mixed‐model formulas of CIE coordinates, conventional D‐A‐type TADF emitters require an extremely deep‐blue emission to meet the BT.2020 blue standard, as their broad spectra may easily cover the adjacent green region and thereby significantly affect the CIE_y_ value. As for MR‐type TADF emitters with narrowband characteristics, a modest blueshift is sufficient to bring their CIE coordinates in close proximity to the BT.2020 blue standard. In this section, two types of blue TADF emitters are both summarized, and strategies to shift the CIE coordinates close to the BT.2020 blue standard are discussed (**Figure** **8**, **Table** **4** **and** **5**).

**Table 4:** PL and EL data of D‐A‐type blue TADF emitters discussed in Section 5.1

Emitter	τ_D_ ^a)^ [µs]	λ_EL_ ^b)^ [nm]	EQE_max/100/1000_ ^c)^ [%]	CE_max_ ^d)^ [cd A^−1^]	PE_max_ ^e)^ [lm W^−1^]	Lifetime [h]	CIE (x, y)^f)^	Reference
tBuCz‐DPS(3)	‐	425	9.9/2.1/‐	‐	‐	‐	(0.15, 0.07)	[81]
DCzBN1	18	418	2.5/‐/‐	0.8	0.6	‐	(0.15, 0.05)	[82]
DCzBN2	11.2	436	7.7/‐/‐	3.9	2.7	‐	(0.15, 0.07)	[82]
DCzBN3	13.5	428	10.3/‐/‐	5.1	3.5	‐	(0.16, 0.06)	[82]
5CzPhCF_3_	0.14	431	9.5/‐/‐	1.4	0.78	‐	(0.157, 0.066)	[83]
CNBuCz‐TRZ	‐	434	4.1/2.0/‐	2.5	1.9	‐	(0.157, 0.063)	[86]
DCBTRZ	0.1	440	6.7/4.5/3.6	3.4	3.0	‐	(0.153, 0.059)	[87]
PPCTRZ	0.3	442	6.6/5.2/4.5	3.8	3.8	‐	(0.153, 0.063)	[87]
12AcCz‐PM	‐	433	3.4/‐/‐	1.4	0.6	‐	(0.15, 0.05)	[89]
TDBA‐Ac	‐	448	21.5/18.2/10.0	8.5	7.6	LT_50_ (500 cd m^−2^) < 2	(0.15, 0.06)	[91]
TDBA‐PAS	2.5	435	22.4/18.0/‐	9.0	5.6	‐	(0.155, 0.042)	[92]
sAc‐sDBB	134	444	25.4/24.6/20.0	14.8	13.7	‐	(0.151, 0.058)	[93]
sAc‐DBB	106	430	15.4/13.4/11.1	8.1	7.1	‐	(0.155, 0.047)	[93]

^a)^ Delayed lifetime in film state

^b)^ peak wavelength of EL spectrum

^c)^ maximum external quantum efficiency, external quantum efficiency at 100 cd m^−2^, and external quantum efficiency at 1000 cd m^−2^

^d)^ maximum current efficiency

^e)^ maximum power efficiency

^f)^ CIE coordinates of EL spectrum.

**Table 5:** PL and EL data of MR‐type blue TADF emitters discussed in Section 5.2

Emitter	PL data			EL data								Reference
	λ_PL_ ^a)^ [nm]	FWHM^b)^ [nm]	τ_D_ ^c)^ [µs]	Sensitizer	λ_EL_ ^d)^ [nm]	FWHM^e)^ [nm]	EQE_max/100/1000_ ^f)^ [%]	CE_max_ ^g)^ [cd m^−1^]	PE_max_ ^h)^ [lm W^−1^]	Lifetime [h]	CIE (x, y) ^i)^	
DABNA‐1	462^j)^	33^j)^	93.7	n/a	459	28	13.5/6.2/‐	10.6	8.3		(0.13, 0.09)	[21]
*t*‐DAB‐DPA	451	24	28.8	n/a	459	26	27.9/21.8/8.1	‐	‐		(0.13, 0.08)	[94]
BFCz‐DABNA	456	22	18.5	n/a	463	26	28.0/13.4/5.1	23.1	‐		(0.13, 0.09)	[97]
pBP‐DABNA‐Me	462	22	53	n/a	464	23	23.4/20.0/3.2	17.4	‐		(0.132, 0.092)	[98]
C‐BN	437	19	‐	p4TzPhBN	453^k)^	25^k)^	26.6/20.1/8.9^k)^	‐	20.1^k)^	LT_90_ (500 cd m^−2^) = 12.3^k)^	(0.14, 0.07)^k)^	[99]
BN1	454	18	127	3Cz2BN	457^k)^	28^k)^	31.2/18.3/9.3^k)^	20.9^k)^	14.9^k)^		(0.14, 0.08)^k)^	[100]
BN3	456	17	17.8	3Cz2BN	458^k)^	23^k)^	37.6/34.0/26.2^k)^	27.5^k)^	19.1^k)^	LT_50_ (100 cd m^−2^) = 27.2^k)^	(0.14, 0.08)^k)^	[100]
B‐O‐dpa	433	28	224	n/a	443	32	16.3/2.2/‐	8.3	‐	LT_50_ (10 cd m^−2^) = 3.5	(0.15, 0.05)	[101]
CzBNO	443	23	48.0	n/a	454	36	13.6/10.1/5.5	14.7	‐	LT_50_ (100 cd m^−2^) = 0.4	(0.14, 0.08)	[102]
CzBO	445	26	120	n/a	448	30	13.4/8.4/3.5	7.4	5.7	LT_50_ (100 cd m^−2^) = 0.2	(0.15, 0.05)	[103]
1B‐DTACrs	438	27	‐	n/a	440	30	1.3/‐/‐	‐	‐	‐	(0.154,0.049)	[104]
2B‐DTACrs	443	21	13.1	n/a	447	26	14.8/6.2/‐	‐	‐	‐	(0.150, 0.044)	[104]
DIDOBNA‐N	426	434	13.7	n/a	429	70	15.2/3.8/‐	‐	6.5	‐	(0.152, 0.073)	[105]
Mes‐BDIDOBNA‐N	399	23	95.9	n/a	402	22	9.3/‐/‐	‐	1.1	‐	(0.165, 0.044)	[105]
NOBNacene	405^l)^	40^l)^	1180	n/a	409	37	8.5/‐/‐	‐	2.1	‐	(0.173, 0.055)	[106]
*v*‐DABNA	468	14	4.1	n/a	469	18	34.4/32.8/26.0	31.0	25.6	LT_50_ (100 cd m^−2^) = 31	(0.12, 0.11)	[22]
*v*‐DABNA‐O‐Me	461	19	7.7	n/a	465	18	29.5/28.8/26.9	24.6	22.7	LT_50_ (100 cd m^−2^) = 314	(0.13, 0.10)	[107]
BOBO‐Z	441	15	7.7	n/a	445	18	13.6/9.8/3.3	7.2	5.0	LT_50_ (100 cd m^−2^) = 0.2	(0.15, 0.04)	[108]
BOBS‐Z	453	21	7.6	n/a	456	23	26.9/24.0/15.0	16.7	12.9	LT_50_ (100 cd m^−2^) = 1.1	(0.14, 0.06)	[108]
BSBS‐Z	460	20	6.7	n/a	463	22	26.8/24.0/15.9	23.2	15.0	LT_50_ (100 cd m^−2^) = 1.2	(0.13, 008)	[108]
TPD4DPA	445	19	4.7	n/a	455	29	30.7/30.6/17.8	15.7	‐	‐	(0.14, 0.06)	[109]
tBu‐TPAD4DPA	451	19	5.6	n/a	460	29	32.5/30.9/20.5	19.4	‐	‐	(0.14, 0.07)	[109]
4F‐ν‐DABNA	457	14	3.1	n/a	464	18	35.8/26.4/10.2	26.8	‐	‐	(0.13, 0.08)	[110]
4F‐m‐ν‐DABNA	455	14	3.2	n/a	461	18	33.7/25.8/‐	24.9	‐	‐	(0.13, 0.06)	[110]
V‐DABNA‐F	467	13	1.9	n/a	468	15	26.6/25.8/23.4	22.3	21.5	LT_50_ (500 cd m^−2^) = 3.6	(0.12, 0.10)	[111]
CZCO	410	32	393	n/a	432	35	15.6/6.1/2.9	8.6	7.1	‐	(0.157, 0.071)	[112]
CZ2CO	434	16	432	n/a	445	23	13.1/9.2/5.8	9.4	5.9	‐	(0.154, 0.051)	[112]

^a)^ Peak wavelength of PL spectrum in dilute toluene solution

^b)^ full width at half maximum of PL spectrum

^c)^ delayed lifetime in film state

^d)^ peak wavelength of EL spectrum

^e)^ full width at half maximum of EL spectrum

^f)^ maximum external quantum efficiency, external quantum efficiency at 100 cd m^−2^, and external quantum efficiency at 1000 cd m^−2^

^g)^ maximum current efficiency

^h)^ maximum power efficiency

^i)^ CIE coordinates for EL spectrum

^j)^ in dilute dichloromethane solution

^k)^ HF(TADF‐sensitized) OLED

^l)^ in dilute tetrahydrofuran solution.

### 5.1 Conventional D‐A‐Type Blue TADF Emitters

**Figure 9:**
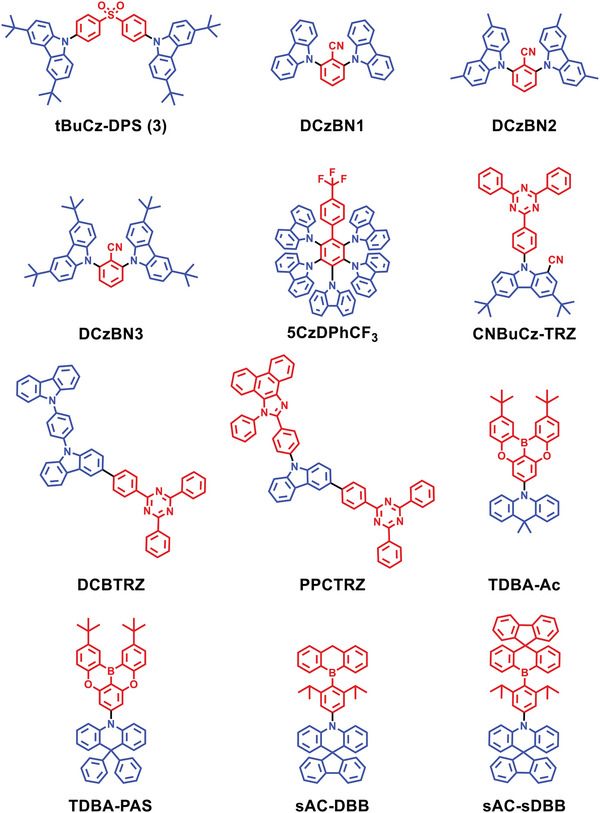
Molecular structures of D‐A‐type blue TADF emitters discussed in Section 5.1.

A blue to deep‐blue emission corresponds to a wide optical bandgap, which can be realized by adopting weak electron‐donating and electron‐accepting units in D‐A‐type emitters. However, it is rather difficult to realize blue, especially deep‐blue, TADF emissions, as the critical CT effect in such cases is often not apparent, leading to suboptimal or absent TADF characteristics. The current reported D‐A type TADF emitters with CIE coordinates approaching the BT.2020 blue standard mainly rely on dynamic incorporation of electron‐donating/accepting pairs, such as Cz‐derived electron‐donating units–moderated electron‐accepting units or acridine‐derived electron‐donating units–weak electron‐accepting units (**Figure** **9**).

In 2012, Zhang and coworkers reported a deep‐blue emitter **tBuCz‐DPS** with 3,6‐di‐tertbutylcarbazole (tBuCz) as the donor and diphenylsulfon (DPS) as the acceptor.^[^ ^81^ ^]^ **tBuCz‐DPS** exhibits a deep‐blue emission with peak at ≈405 nm. However, due to the lower local excited triplet energy level of tBuCz than the combined CT one, **tBuCz‐DPS** shows a large ΔE_ST_ value of 0.32 eV, thus leading to poor TADF characteristics. Therefore, to attain efficient deep‐blue TADF emissions, both redox potential and local excited triplet energy levels of the donor and acceptor moieties should be taken into account. The deep‐blue OLED based on **tBuCz‐DPS** achieves a decent EQE_max_ of 9.9% and CIE coordinate of (0.15, 0.07). This report demonstrated the basic design principle of blue to deep‐blue TADF emitters.

In 2018, Adachi and coworkers reported a series of deep‐blue TADF emitters with Cz as the donor and benzonitrile as the acceptor.^[^ ^82^ ^]^ By simple modification of the functional groups on the donor unit, these emitters not only realize tunable deep‐blue emission with peaks at 410, 417, and 414 nm, but also show improved TADF properties with ΔE_ST_ values of 0.31, 0.22, and 0.26 eV, for **DCzBN1**, **DCzBN2**, and **DCzBN3**, respectively. The corresponding OLEDs exhibit deep‐blue EL with λ_EL_s at 418, 436, and 428 nm, CIE coordinates of (0.15, 0.05), (0.15, 0.07) and (0.16, 0.07), and EQE_max_s of 2.5%, 7.7%, and 10.3% for **DCzBN1**, **DCzBN2**, and **DCzBN3**, respectively. This category of deep‐blue emitters has the potential to serve as assistant dopants in hyperfluorescence (HF, i.e., TADF‐sensitized) systems, offering a promising approach towards stable blue OLEDs. To further advance the development of deep‐blue TADF emitters based on multiple Cz donors, Tao and coworkers reported a deep‐blue emitter **5CzDPhCF_3_** by simply introducing a phenyl bridge between the Cz donors and the CF_3_ acceptor.^[^ ^83^ ^]^ The π‐bridge significantly reduces the electron‐accepting ability of the nonconjugated CF_3_ acceptor, leading to blueshifted emission with a peak at 431 nm in dilute toluene solution. The **5CzDPhCF_3_**‐based OLED exhibits deep‐blue EL with an EQE_max_ of 9.5% and CIE coordinates of (0.157, 0.066).

Earlier studies have demonstrated that the 2,4,6‐triphenyltriazine (TRZ) acceptor exhibits favorable compatibility with the Cz donor in constructing efficient blue TADF emitters.^[^ ^84^ ^]^ However, such a TRZ‐Cz framework generally exhibits blue to sky‐blue emission in OLEDs, which falls short of meeting the BT.2020 blue standard.^[^ ^85^ ^]^ To blueshift the CIE coordinates, Wang and coworkers proposed a donor‐modification strategy and developed a series of blue to deep‐blue TADF emitters by introducing an electron‐accepting unit into the 1‐position of Cz.^[^ ^86^ ^]^ The substituent units introduced at the 1‐position lead to blueshifted emissions of these emitters. In particular, **CNBuCz‐TRZ** with the incorporation of a cyan group exhibits a deep‐blue emission peaking at 398 nm in dilute toluene solution. The OLED fabricated with **CNBuCz‐TRZ** exhibits deep‐blue EL with λ_EL_ at 434 nm and CIE coordinates of (0.157, 0.063). Most recently, Zhao and coworkers reported two deep‐blue TADF emitters, **DCBTRZ** and **PPCTRZ,** by directly using the host materials DCB and PPC as the donors and TRZ as the acceptor.^[^ ^87^ ^]^ Owing to the electron extension of the HOMOs, **DCBTRZ** and **PPCTRZ** not only possess decent TADF characteristics but also exhibit deep‐blue emission with peaks at 428 and 430 nm. It is noteworthy that **DCBTRZ** and **PPCTRZ** also reveal narrowband features with FWHMs of 42 nm, which are comparable to MR‐type emitters. The OLEDs based on **DCBTRZ** and **PPCTRZ** exhibit similar deep‐blue EL with λ_EL_s at 440 and 442 nm, FWHMs of 56 and 54 nm, CIE coordinates of both (0.15, 0.06), and EQE_max_s of 6.9% and 6.6%, respectively.

The acridine derivatives possess a more potent electron‐donating ability than their Cz counterpart, necessitating the incorporation of weak electron‐accepting moieties to achieve blue or deep‐blue emission.^[^ ^88^ ^]^ In 2019, Wang and coworkers reported a deep‐blue emitter **12AcCz‐PM** with 13,13‐dimethyl‐7‐phenyl‐7,13‐dihydro‐5*H*‐indolo[3,2‐*b*]acridine (12AcCz) as the donor and 2,4,6‐triphenylpyrimidine (TPPM) as the acceptor.^[^ ^89^ ^]^ The large steric hindrance of indole fused in the 1,2‐position of 12AcCz‐PM compelled **12AcCz‐PM** to form a quasi‐axial conformation, leading to a blueshifted emission with a peak at ≈440 nm. The optimal OLED based on **12AcCz‐PM** presents deep‐blue EL with λ_EL_ at 438 nm, CIE coordinates of (0.15, 0.05), and an EQE_max_ of 5.7%.

In recent years, cyclized boron‐based acceptors have garnered favorable attention in constructing efficient blue TADF emitters due to their appropriate LUMO energy levels and high performance in OLEDs.^[^ ^90^ ^]^ By employing oxygen‐bridged triarylboron as the acceptor, Kwon and coworkers reported two efficient blue TADF emitters.^[^ ^91^ ^]^ Benefiting from the narrowband feature of such an acceptor, the bluer emitter **TDBA‐Ac** exhibits pure‐blue emission with a peak at 458 nm and an FWHM of 50 nm in dilute toluene solution. Consequently, the OLED with **TDBA‐Ac** as the emitter presents pure‐blue EL with a peak at 448 nm, an FWHM of 48 nm, and CIE coordinates of (0.15, 0.06). Moreover, the device achieves a high EQE_max_ of 21.5%. Notably, the OLED with the less blue emitter **TDBA‐DI** as the emitter demonstrates a record‐high EQE_max_ of 38.2% with relieved efficiency roll‐off. Based on this report, Tong and coworkers developed a deep‐blue TADF emitter **TDBA‐PAS** with conformational heterogeneity by introducing sp^3^‐Si‐based acridine as donor.^[^ ^92^ ^]^ **TDBA‐PAS** exhibits dual fluorescence emission with two peaks at 338 and 427 nm. Furthermore, the presence of quasi‐axial conformations plays a crucial role in mitigating concentration quenching of emitted quasi‐equatorial conformations, resulting in **TDBA‐PAS** exhibiting consistent PLQYs above 80% across doping ratios ranging from 10 to 30 wt.%. Consequently, **TDBA‐PAS**‐based OLEDs achieve high EQE_max_s of ≈20%, with reduced efficiency roll‐off and CIE coordinates of ≈ (0.15, 0.04) close to the Rec.2020 blue standard over a wide doping range of 10–50 wt.%. This study highlights a new strategy for mitigating spectral redshift and concentration quenching in the design of deep‐blue TADF emitters.

Similarly, to resolve the lack of highly efficient deep‐blue OLEDs, Wang and coworkers reported a molecular design strategy of incorporating weak spiro‐donor and spiro‐acceptor groups into a linear D‐π‐A type molecule through a sterically bulky p‐spacer.^[^ ^93^ ^]^ Owing to their high molecular rigidity, the target emitters **sAC‐sDBB** and **sAC‐DBB** both exhibit deep‐blue narrowband emissions with peaks at 453 and 455 nm and FWHMs of 51 and 54 nm. Compared to **sAC‐DBB**, the steric hindrance of the terminal spiral fragments in **sAC‐sDBB** contributes to the better suppression of concentration quenching, thus offering **sAC‐sDBB** superior photophysical properties, i.e., high PLQYs of above 80% and maintaining narrowband spectra in both doped and neat films. Consequently, the **sAC‐sDBB**‐based OLED achieves a high EQE_max_ of 25.4% and shows narrowband deep‐blue EL with a peak at 444 nm, an FWHM of 49 nm, and CIE coordinates of (0.151, 0.058). For the OLED based on **sAC‐DBB**, narrowband deep‐blue EL with a lower EQE_max_ of 15.4% and CIE coordinates of (0.155, 0.047) was observed. Notably, the nondoped OLED based on **sAC‐sDBB** demonstrates an impressive EQE_max_ of 22.5% with CIE_y_ <0.1, representing the record‐high EQE value among the deep‐blue nondoped OLEDs with CIE_y_ <0.1 ever reported. These results further indicate the superiority of cyclized boron‐based acceptors in constructing deep‐blue D‐A‐type TADF emitters.

### 5.2 MR‐Type Blue TADF Emitters

**Figure 10:**
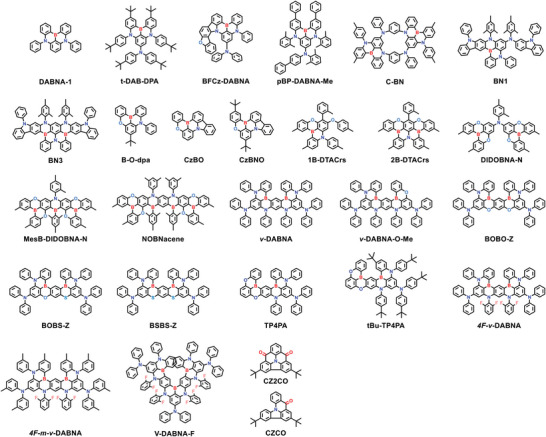
Molecular structures of MR‐type blue TADF emitters discussed in Section 5.2.

The exceptional narrowband feature, well‐induced TADF characteristics, and natural blue optical bandgap of MR‐type frameworks permit MR‐type emitters to be ideal candidates for the BT.2020 blue standard (**Figure** **10**). As pioneers in the field of these narrowband materials, Hatakeyama and coworkers developed the first narrowband deep‐blue MR‐type TADF emitters **DABNA‐1** and **DABNA‐2** by incorporating one B and two N atoms into the same polycyclic aromatic framework.^[^ ^21^ ^]^ Owing to the desirable MR effect induced by *ortho*‐disposed B/N atoms, the HOMO and LUMO of **DABNA‐1** and **DABNA‐2** are alternately distributed, resulting in efficient TADF characteristics with small ΔE_ST_ values of 0.18 and 0.14 eV, respectively. Impressively, **DABNA‐1** and **DABNA‐2** display pure‐blue narrowband emissions with peaks at 462 and 470 nm, and considerably narrow FWHMs of 33 and 34 nm in dilute dichloromethane solution. Consequently, the **DABNA‐1**‐based OLED exhibits a pure‐blue EL with peak at 459 nm, FWHM of 28 nm, and CIE coordinates of (0.13, 0.09), while the **DABNA‐2**‐based OLED exhibits a slightly redshifted EL with a peak at 467 nm, the same FWHM of 28 nm, and CIE coordinates of (0.12, 0.13). In addition, the proposed “one‐pot borylation” can precisely introduce B atoms into the carbon (C)–hydrogen (H) sites with the assistance of lithiation, which allow for the sufficient design flexibility of boron‐embedded MR‐TADF emitters. Overall, this work sparks significant interest among researchers and establishes the groundwork for the advancement of such MR‐type blue emitters.

In order to meet the BT.2020 blue standard, it is imperative to further blueshift the emission spectrum of **DABNA‐1**. A straightforward approach involves the modification of the electron‐donating group at the *para*‐position of the B atom, which serves to mitigate the electron‐withdrawing capacity of the whole MR skeleton, thus leading to a blueshifted emission without spectral broadening. In 2021, Lee and coworkers reported a deep‐blue MR‐type emitter ***t*‐DAB‐DPA** by introducing an additional bis(*tert‐*butylphenyl)amine (*t*DPA) group at the *para*‐position of the B atom.^[^ ^94^ ^]^ Compared with the parent **DABNA‐1**, ***t*‐DAB‐DPA** displays slightly blueshifted narrowband emission with a peak at 451 nm and a small FWHM of 24 nm in dilute toluene solution. Notably, the induction of the *t*DPA group can also minimize the effect of the intermolecular interaction and surrounding environment, thus leading to suppressed concentration quenching. The OLEDs based on ***t*‐DAB‐DPA** achieve high EL performance with decent EQEs of 27.6%, 27.9%, 27.4%, and 25.9% at 3, 5, 7, and 10 wt.% doping concentrations, respectively. Furthermore, these devices also achieve deep‐blue CIE coordinates of (0.13, 0.08) and narrow FWHMs of 26 nm without concentration‐dependent spectral shifting or broadening. Notably, the MR emitter with the same molecular structure as ***t*‐DAB‐DPA** were also reported by Hatakeyama et al. and Wang et al., respectively named **DABNA‐NP‐TB** and **3tPAB**.^[^ ^95^, ^96^ ^]^ In the work of Hatakeyama et al., they also revealed the reactivity and regioselectivity of one‐shot borylation by density functional theory calculations, offering valuable insights into the synthetic methodology for boron‐embedded MR‐TADF emitters. Following above reports, Kwon and coworkers reported a Cz‐fused emitter **BFCz‐DABNA** by incorporating a weak electron‐donating benzofurancarbazole (BFCz) moiety.^[^ ^97^ ^]^ The introduction of the BFCz moiety significantly enhances the molecular rigidity, thus resulting in narrower deep‐blue emission with a small FWHM of only 22 nm in dilute toluene solution. The corresponding OLED with **BFCz‐DABNA** as the emitter achieves a high EQE_max_ of 28.0% with deep‐blue CIE coordinates of (0.13, 0.09). To further enhance the performance of such emitters, Kim and coworkers in 2022 developed a deep‐blue MR‐type TADF emitter ***p*BP‐DABNA‐Me** by introducing biphenyls and N‐biphenyl‐N‐orthodimethylphenylamine substituents.^[^ ^98^ ^]^ The substituents not only serve to balance the push‐pull effect in the optical bandgap, leading ***p*BP‐DABNA‐Me** to maintain a deep‐blue emission with a peak at 462 nm and an FWHM of 22 nm in dilute toluene solution, but also create dense local triplet states and suppress intramolecular aggregation. It is noteworthy that local triplet states possess a strong SOC effect with the emissive S_1_ state, which can lead to a highly enhanced RISC process. The OLED based on ***p*BP‐DABNA‐Me** achieves an optimized EQE_max_ of 23.4% and exhibits pure‐blue EL with CIE coordinates of (0.132, 0.092). Notably, the pure‐blue EL is maintained even in a nondoped OLED. Furthermore, by incorporating the conventional TADF emitter TDBA‐SAF as an assistant sensitizer, the device demonstrates an improved EQE_max_ of 30.1%. This work provides insight into design strategies for developing efficient deep‐blue MR‐type TADF emitters with fast triplet upconversion and suppressed self‐aggregation. In addition, Duan and coworkers reported a macrocycle of B/N‐doped calix[4]arene (**C‐BN**) based on the parent skeleton of ***t*‐DAB‐DPA**.^[^ ^99^ ^]^ **C‐BN** exhibits deep‐blue emission with a peak at 437 nm and an FWHM of 19 nm. The OLED fabricated with **C‐BN** achieves an EQE_max_ of 20.1% and deep‐blue CIE coordinates of (0.14, 0.07). In the same year, Yang and coworkers reported a deep‐blue MR‐TADF emitter **BN1** featuring an enlarged ring‐fused structure, along with this double B‐embedded counterpart **BN3**.^[^ ^100^ ^]^ Notably, these compounds were obtained by one‐shot borylation from the same electron‐rich precursor in high yields at different conditions (i.e., dynamically control the reaction temperature and the equivalent of boron tribromide).With the DPA derivatives at the *para‐*position of the B atom, **BN1** exhibits a deep‐blue emission with a peak at 454 nm and a small FWHM of 18 nm in dilute toluene solution. It is worth noting that the molecular structural extension enhances the TADF characteristic of **BN3**, resulting in a reduced ΔE_ST_ value of 0.15 eV and an increased k_RISC_ rate up to 2.6×10^5^ s^−1^, while maintaining deep‐blue emission with a peak at 456 nm and a narrow FWHM of only 17 nm. By employing a blue TADF emitter **3Cz2BN** as an assistant sensitizer, the **BN‐3**‐based OLED achieves a record‐high EQE_max_ of 37.6% and pure‐blue EL with λ_EL_ at 458 nm, an FWHM of 23 nm and CIE coordinates of (0.14, 0.08).

As mentioned in Section 2, spectral blueshift can also be attained through the substitution of N atoms with O atoms in the MR skeletons. In 2021, Lee and coworkers reported a series of blue MR‐type TADF emitters with an asymmetric molecular structure containing B, N, and O atoms.^[^ ^101^ ^]^ Notably, the borylation of these emitters were synthesized via Lewis‐acid‐mediated cyclization from pinacolato‐borate‐based precursors. These emitters all exhibit blue narrowband emissions due to the incorporation of weak electron‐donating O atoms, where the deep‐blue emitter **B‐O‐dpa** displays narrowband emission with a peak at 433 nm and FWHM of 28 nm in dilute toluene solution. The OLED based on **B‐O‐dpa** exhibits deep‐blue EL with a peak at 443 nm, an FWHM of 32 nm, CIE coordinates of (0.15, 0.05) and EQE_max_ of 16.3%. The deep‐blue emitters **CzBNO** and **CzBO** with similar Cz‐fused molecular structures have been reported in subsequent work by Yang et al. and Yasuda et al., respectively.^[^ ^102^, ^103^ ^]^ The corresponding OLEDs exhibit deep‐blue EL with EQE_max_s of 13.6% and 13.4% and CIE coordinates of (0.14, 0.08) and (0.15, 0.05) for **CzBNO** and **CzBO**, respectively. Subsequently, Eli and coworkers reported a series of O‐modified blue MR‐type emitters that gradually increase in the number of embedded B atoms from one to four.^[^ ^104^, ^105^, ^106^ ^]^ The corresponding OLEDs achieve high EQE_max_s of 1.3%, 14.8%, 15.2%, 9.3% and 8.5%, and CIE coordinates of (0.154, 0.049), (0.150, 0.044), (0.152, 0.073), (0.165, 0.044) and (0.173, 0.055) for **1B‐DTACrs**, **2B‐DTACrs**, **DIDOBNA‐N**, **Mes‐BDIDOBNA‐N**, and **NOBNacene**, respectively.

Early in 2019, Hatakeyama and coworkers reported an upgraded version of **DABNA‐1**, named ***ν*‐DABNA**, which consists of five benzene rings connected by two B atoms, four N atoms, and two DPA groups.^[^ ^22^ ^]^ ***v*‐DABNA** was synthesized via a direct electrophilic C‐H borylation, i.e., one‐shot borylation, with a detected yield of ≈73%. This method allowed for high synthetic yields without requiring an intermediate lithiation process, further accelerating the development of multiple‐boron‐embedded MR‐TADF emitters. The incorporation of multiple B/N atoms in ***ν*‐DABNA** significantly enhances the MR effect, leading to the localization of HOMO and LUMO on different atoms and minimizing their bonding/antibonding character; nonbonding molecular orbitals are thus formed in ***ν*‐DABNA**, which minimize vibronic coupling and vibrational relaxation, resulting in an extremely sharp blue emission with an FWHM of only 14 nm in dilute toluene solution. The OLED fabricated with ***ν*‐DABNA** exhibits an ultranarrow blue EL with λ_EL_ at 469 nm, FWHM of 18 nm, and CIE coordinates of (0.12, 0.11), which is comparable to the latest well‐defined LEDs. In addition, the device also achieves a considerably high EQE_max_ of 34.4% with suppressed efficiency roll‐off (e.g., EQE of 26.0% at 1000 cd m^−2^). Owing to its remarkable OLED performance, ***v*‐DABNA** is now recognized as a pinnacle for blue MR‐type emitters and serves as a basic blue MR template for structural modification. Subsequently, the same group reported an ultrapure deep‐blue MR‐type emitter ***ν*‐DABNA‐OMe** by adopting an O‐atom‐incorporation strategy in ***ν*‐DABNA**.^[^ ^107^ ^]^ Compared to ***ν*‐DABNA**, the incorporation of one O atom allows ***ν*‐DABNA‐OMe** to display deeper blue emission with a peak at 461 nm and an FWHM of 19 nm in dilute toluene solution. The OLED based on ***ν*‐DABNA‐OMe** exhibits pure‐blue EL with λ_EL_ at 465 nm, FWHM of 18 nm, CIE coordinates of (0.13, 0.10) and a high EQE_max_ of 29.5%. Similarly, Yasuda and coworkers reported three ultrapure blue MR‐type emitters, **BOBO‐Z**, **BOBS‐Z**, and **BSBS‐Z**, by systematically replacing two N atoms of ***ν*‐DABNA** with O and/or sulfur (S) atoms.^[^ ^108^ ^]^ Because of weaker electron‐donating abilities of O/S than N atoms, bluer narrowband emission in dilute toluene solution with peaks at 441, 453, and 460 nm, and FWHMs of 15, 21, and 20 nm were observed for **BOBO‐Z**, **BOBS‐Z**, and **BSBS‐Z**, respectively. Notably, the S‐doped emitters **BOBS‐Z** and **BSBS‐Z** exhibit gradually enhanced *k*_RISC_ as the number of S atoms increases due to the heavy‐atom effect. Consequently, the corresponding OLEDs demonstrate ultrapure blue EL with decent EQE_max_s of 13.6%, 26.9%, and 26.8%, λ_EL_ at 445, 456, and 463 nm, FWHMs of 18, 23 and 22 nm and CIE coordinates of (0.15, 0.04), (0.14, 0.06), and (0.13, 0.08) for **BOBO‐Z**, **BOBS‐Z**, and **BSBS‐Z**, respectively. Most recently, Kwon and coworkers reported two deep blue MR‐TADF emitters, **TPD4PA** and **tBu‐TPD4PA**, by incorporating two O atoms on one side of ***v*‐DABNA**.^[^ ^109^ ^]^ **TPD4PA** and **tBu‐TPD4PA** exhibit deep‐blue narrowband emissions with peaks at 445 and 451 nm and FWHMs of both 19 nm in dilute toluene solution. The corresponding OLEDs achieve high EQE_max_s of 30.7% and 32.5% and deep‐blue EL with CIE coordinates of (0.14, 0.06) and (0.15, 0.07) for **TPD4PA** and **tBu‐TPD4PA**, respectively.

The introduction of fluorine (F) atoms represents another effective approach to inducing a blueshift in the CIE coordinates of ***v*‐DABNA**. In 2022, Kwon and coworkers reported two deep‐blue MR‐type emitters, **4F‐ν‐DABNA** and **4F‐m‐ν‐DABNA**, by introducing F atoms at the *ortho*‐positions of the N atoms.^[^ ^110^ ^]^ The electronegative F atoms exert an attenuating effect on the electron‐donating ability of the corresponding N atoms, leading to deeper blue emission with peaks at 457 and 455 nm for **4F‐ν‐DABNA** and **4F‐m‐ν‐DABNA**, respectively. The corresponding OLEDs achieve considerably high EQE_max_s of 35.8% and 33.7% and present deep‐blue EL with peaks at 464 and 461 nm, FWHMs of both 18 nm, and CIE coordinates of (0.13, 0.08) and (0.13, 0.06), for **4F‐ν‐DABNA** and **4F‐m‐ν‐DABNA**, respectively. Based on this report, Hatakeyama and coworkers developed a large‐size version, named **V‐DABNA‐F**, via one‐shot triple borylation with a decent yield.^[^ ^111^, ^113^ ^]^ **V‐DABNA‐F** shows a pure‐blue narrowband emission with a peak at 467 nm and an FWHM of 13 nm in dilute toluene solution. The corresponding OLED with V‐DABNA‐F as the emitter exhibits pure‐blue EL with a peak at 468 nm, an FWHM of 15 nm, CIE coordinates of (0.12, 0.10) and EQE_max_ of 26.6%.

In addition, Lee and coworkers developed two deep‐blue **CZCO** and **CZ2CO** emitters based on the reported N/carbonyl MR skeleton **QAD**.^[^ ^112^, ^114^, ^115^, ^116^ ^]^ The cyclization of **CZ2CO** serves to enhance the molecular rigidity, strengthen the resonant strength, inhibit molecular bending and rocking, and destabilize the HOMO energy level, leading to deep‐blue narrowband emission with a peak at 440 nm and a small FWHM of 16 nm in dilute toluene solution. The contrast emitter **CzCO** exhibits broader deep‐blue emission with a peak at 440 nm and a larger FWHM of 32 nm. Consequently, the corresponding OLEDs achieve decent EQE_max_s of 15.6% and 13.0% and deep‐blue CIE coordinates of (0.154, 0.047) and (0.154, 0.061) for **CzCO** and **Cz2CO**, respectively. Furthermore, by adopting an HF emitting layer, the **CZCO**‐ and **Cz2CO**‐based devices demonstrate improved EQE_max_ of up to 26.9% and 25.6%, along with mitigated efficiency roll‐offs.

## 6 Summary and Outlook

In pursuit of the wide‐color‐gamut BT.2020 standard for ultra‐high‐resolution OLED displays, it is of great significance to develop high‐standard primary RGB emitters. With the prospect of realizing 100% exciton utilization without resorting to scarce noble metals, significant endeavors have been dedicated to the advancement of TADF emitters since 2012. In this review, the latest RGB TADF emitters with CIE coordinates close to the BT.2020 standard are summarized. Rational strategies for molecular design, as well as the resulting photophysical properties and OLED performances, are expounded upon to reveal the underlying mechanisms for shifting the CIE coordinates of both conventional D‐A‐type and MR‐type TADF emitters toward the BT.2020 standard. For D‐A‐type TADF emitters, spectral shifting is the most effective and applicable method for promoting CIE coordinates toward the BT.2020 standard, which can be achieved by dynamic incorporation of diverse donors and acceptors. MR‐type emitters exhibit theoretical advantages over D‐A‐type emitters in approaching the BT.2020 standard; however, their spectral shifting and trade‐off between spectral shifting and broadening remain great challenges.

The edge positions of the red and blue bands in the whole visible spectrum permit both D‐A‐ and MR‐type TADF emitters to shift their CIE coordinates to the BT.2020 red and blue standard by adjusting the spectral wavelengths. Correspondingly, a few advanced red and blue TADF emitters have already been achieved or even exceeded the BT.2020 standard in terms of CIE coordinates. However, challenges still remain in improving their emissive efficiency and efficiency roll‐off, which limit their overall performance, especially for blue TADF emitters. The lower satisfaction in EL performance can be mainly attributed to the inefficient TADF characteristics, which result in increased occurrences of triplet‐related exciton deactivation processes such as triplet‐triplet or singlet‐triplet annihilation. Therefore, exploiting standard‐blue/red TADF emitters capable of fast triplet upconversion should be the main pursuits to improve their EL performance. In addition, emerging sensitized‐OLEDs (e.g., HF, PSTADF), which can significantly enhance the ultimate EL performance by employing additional sensitizers to harvest triplet excitons, need further investigation, including the underlying sensitized‐mechanism, OLED device structures, and sensitizer material systems. The green band falls within the central region of the entire visible spectrum, resulting in a narrower valid range than that of the blue and red bands. This relatively limited spectral range presents a significant challenge for TADF emitters to achieve CIE coordinates approaching the BT.2020 green standard, as even a slight shift in peak wavelength or broadening of spectral width can result in overlapping with adjacent blue or red regions, thereby compromising overall color quality. To date, only a few MR‐type green emitters can surpass the NTSC green standard, however, there is still a discernible gap from the BT.2020 green standard. By referring to state‐of‐the‐art quantum dot (QD)‐LEDs, the standard green EL with CIE coordinates (0.170, 0.797) can be realized with a Gaussian‐distributed amorphous shape with a peak at ≈528 nm and an FWHM below 16.5 nm, which is undoubtfully a plausible goal for MR‐type TADF emitters.^[^ ^117^ ^]^ However, to approach this, it is necessary to make special efforts that go beyond resolving the common challenges faced by blue and red ones as well. First, the underlying mechanism of spectral width and the controlling approach to suppress shoulder‐peak/bottom‐trail need further investigation, e.g., how to control the Huang‐Rhys factor of the vibrational 0‐0 vibronic mode coupled with the electronic S_1_ to S_0_ transition, and how to minimize the contribution of the 0‐n vibronic transition, as both are crucial for the calibration of green CIE coordinates. Second, in addition to the conventional understanding that molecular aggregation inhomogeneity induces a broader emission profile in doped films compared to dilute solutions, the previously overlooked solid‐state solvation is of significant concern. To overcome this, a plausible approach would be to develop advanced host materials possessing ultralow polarity. In addition, as outlined in this review, boron‐embedded MR‐TADF emitters have emerged as the mainstream among the reported RGB TADF emitters approaching the BT.2020 standard while their syntheses often encounter great challenges. Therefore, there is an urgent need to further explore effective synthetic methods for borylation.^[^ ^118^, ^119^ ^]^ Overall, through further efforts, it is undoubted that TADF emitters, especially MR‐type emitters, have bright prospects, serving as key emitters for future ultrawide‐color‐gamut OLED displays that offer stunningly vivid colors with unmatched precision.

## Conflict of Interest

The authors declare no conflict of interest.
